# Cancer stem cells and tumor-associated macrophages as mates in tumor progression: mechanisms of crosstalk and advanced bioinformatic tools to dissect their phenotypes and interaction

**DOI:** 10.3389/fimmu.2025.1529847

**Published:** 2025-02-06

**Authors:** Francesco Verona, Sebastiano Di Bella, Roberto Schirano, Camilla Manfredi, Francesca Angeloro, Giulia Bozzari, Matilde Todaro, Giuseppe Giannini, Giorgio Stassi, Veronica Veschi

**Affiliations:** ^1^ Department of Precision Medicine in Medical, Surgical and Critical Care, University of Palermo, Palermo, Italy; ^2^ Department of Molecular Medicine, University La Sapienza, Rome, Italy; ^3^ Department of Health Promotion Sciences, Internal Medicine and Medical Specialties (PROMISE), University of Palermo, Palermo, Italy; ^4^ Azienda Ospedaliera Universitaria Policlinico “Paolo Giaccone” (AOUP), Palermo, Italy; ^5^ Istituto Pasteur, Fondazione Cenci-Bolognetti, Sapienza University of Rome, Rome, Italy

**Keywords:** cancer stem cells, TAMs, single-cell RNA sequencing (scRNA-seq), spatial transcriptomics, signaling pathway analysis, trajectory analysis

## Abstract

Cancer stem cells (CSCs) are a small subset within the tumor mass significantly contributing to cancer progression through dysregulation of various oncogenic pathways, driving tumor growth, chemoresistance and metastasis formation. The aggressive behavior of CSCs is guided by several intracellular signaling pathways such as WNT, NF-kappa-B, NOTCH, Hedgehog, JAK-STAT, PI3K/AKT1/MTOR, TGF/SMAD, PPAR and MAPK kinases, as well as extracellular vesicles such as exosomes, and extracellular signaling molecules such as cytokines, chemokines, pro-angiogenetic and growth factors, which finely regulate CSC phenotype. In this scenario, tumor microenvironment (TME) is a key player in the establishment of a permissive tumor niche, where CSCs engage in intricate communications with diverse immune cells. The “oncogenic” immune cells are mainly represented by B and T lymphocytes, NK cells, and dendritic cells. Among immune cells, macrophages exhibit a more plastic and adaptable phenotype due to their different subpopulations, which are characterized by both immunosuppressive and inflammatory phenotypes. Specifically, tumor-associated macrophages (TAMs) create an immunosuppressive milieu through the production of a plethora of paracrine factors (IL-6, IL-12, TNF-alpha, TGF-beta, CCL1, CCL18) promoting the acquisition by CSCs of a stem-like, invasive and metastatic phenotype. TAMs have demonstrated the ability to communicate with CSCs via direct ligand/receptor (such as CD90/CD11b, LSECtin/BTN3A3, EPHA4/Ephrin) interaction. On the other hand, CSCs exhibited their capacity to influence immune cells, creating a favorable microenvironment for cancer progression. Interestingly, the bidirectional influence of CSCs and TME leads to an epigenetic reprogramming which sustains malignant transformation. Nowadays, the integration of biological and computational data obtained by cutting-edge technologies (single-cell RNA sequencing, spatial transcriptomics, trajectory analysis) has significantly improved the comprehension of the biunivocal multicellular dialogue, providing a comprehensive view of the heterogeneity and dynamics of CSCs, and uncovering alternative mechanisms of immune evasion and therapeutic resistance. Moreover, the combination of biology and computational data will lead to the development of innovative target therapies dampening CSC-TME interaction. Here, we aim to elucidate the most recent insights on CSCs biology and their complex interactions with TME immune cells, specifically TAMs, tracing an exhaustive scenario from the primary tumor to metastasis formation.

## Cancer stem cells hallmarks and crosstalk with TAMs: an old story new

In this review we revised the literature period of the last twenty years using as main keywords the following: cancer stem cells, stemness, tumor-associated macrophages, metastasis, metastatic niche, hallmark, proliferation, immune evasion, neo-angiogenesis, epithelial-mesenchymal transition, crosstalk, pathways, chemoresistance, therapy resistance, target therapy, preclinical model, clinical model, clinical trial, immunotherapy, stemness, self-renewal, invasion, tumorigenicity, oncogenic pathways, metastasis-associated macrophages, tumor microenvironment, scRNA-seq, spatial transcriptomic, trajectory analysis, stromal cells, extracellular matrix and immune cells.

Cancer stem cells (CSCs) are a small subpopulation within tumor bulk sharing features of normal stem cells, such as self-renewal and plasticity (1). Accordingly, the CSC model introduced the concept of the capability of CSCs to recapitulate the intertumoral heterogeneity, differentiating into various cancer cell phenotypes and, in parallel, guaranteeing their population maintenance (2). Due to their genetic flexibility, CSCs can be involved in different biological aspects such as tumor initiation, proliferation, invasion, migration, and chemoresistance (1). All these pro-tumoral traits underlined the critical role of CSCs in cancer progression and made CSCs a potential target for innovative therapeutic approaches (3). Tumor microenvironment (TME) provides an essential environmental niche necessary for cancer development (4). Among the immune cells that have a central role in orchestrating TME, tumor-associated macrophages (TAMs) represent a plastic immune cell population that drives multiple interactions within the TME, leading the spatiotemporal evolution from primary tumor to metastasis (5). TAMs can establish with CSCs an intricate complex communication in fueling different aspects of cancer progression: i) direct ligand-receptor interaction: TAMs expressing colony-stimulating factor (CSF1) receptor anchors CSC-derived CSF1, in the promotion of TAM survival and activation (6); ii) indirect interaction: TAMs release chemokines like chemokine (C–C motif) ligand 2 (CCL2), interleukin-6 (IL-6), interleukin-12 (IL-12), tumor necrosis factor-alpha (TNF-alpha), transforming growth factor-beta-1 (TGFB1) (7); TAMs release exosomes containing microRNAs and proteins that regulate CSC behavior by enhancing stemness and chemoresistance; conversely, CSC-derived exosomes can polarize TAM toward a tumor-promoting M2 phenotype (8, 9).

**Figure 1:**
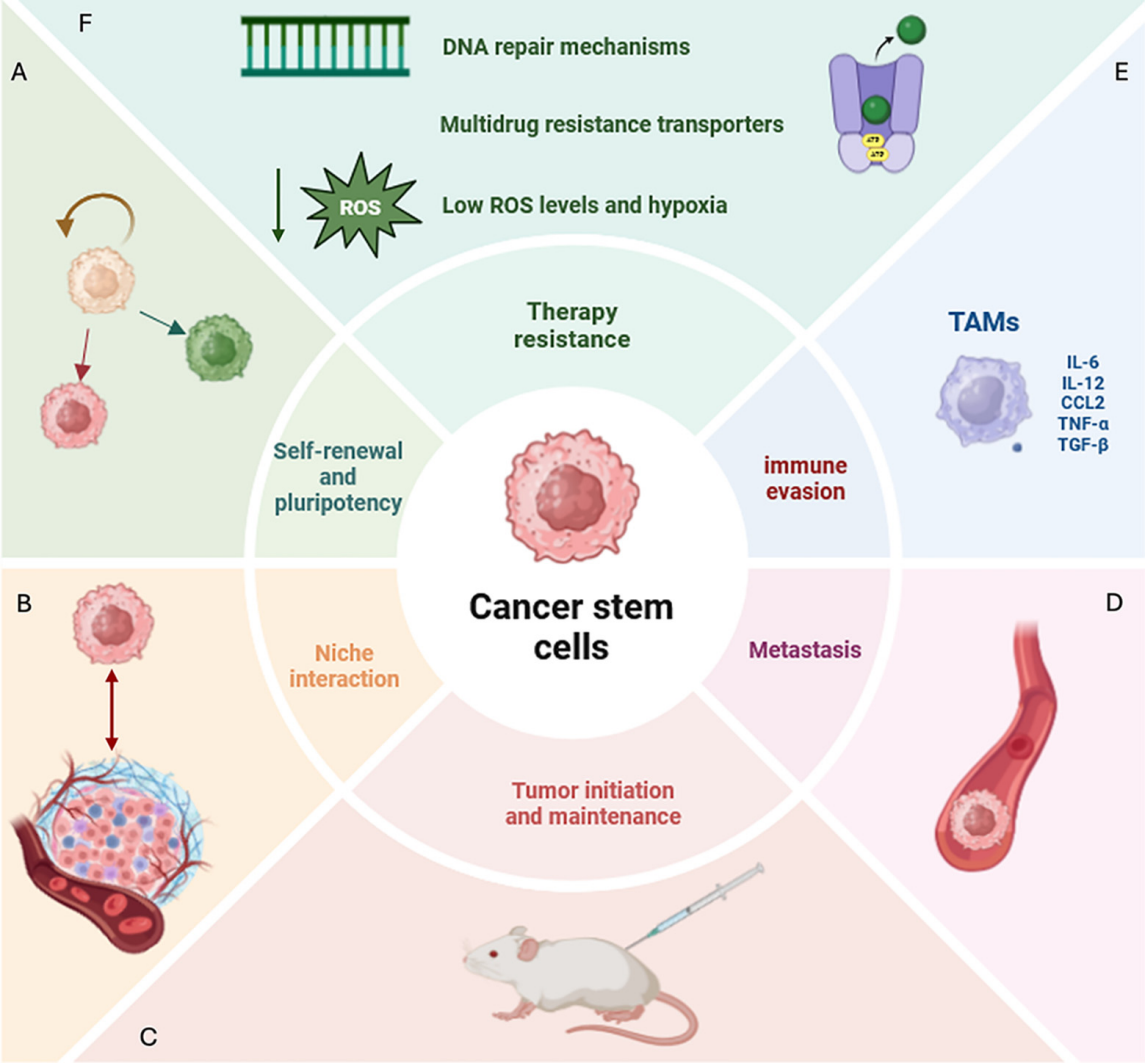
Defining CSC features and hallmarks. **(A)** CSCs (cancer stem cells) display the ability of self-renewal and pluripotency, disrupting tissue homeostasis and generating diverse lineages within the tumor. **(B)** CSCs create a niche in the tumor microenvironment (TME) with which they interact and that proliferates independently of the surrounding tissue. **(C)** CSCs show the ability to initiate tumor growth in immunocompromised mice. **(D)** CSCs represent the most aggressive tumor subpopulation able to spread and form metastases even at distant sites. **(E)** Among immune cells that create an immunosuppressive milieu in CSC-associated TME, in this review we will focus on tumor-associated macrophages (TAMs) which play a critical role. TAMs are macrophages characterized by both immunosuppressive and inflammatory phenotypes. Specifically, they produce a plethora of paracrine factors (IL-6, IL-12, TNF-alpha, TGFB1, CCL2) inducing the acquisition of a stem-like, invasive and metastatic phenotype in CSCs. **(F)** Several mechanisms contributing to therapy resistance in CSCs have been identified, including efficient DNA repair machinery, multidrug resistance transporters, low levels of reactive oxygen species (ROS) and hypoxia. CSCs, cancer stem cell; TME, tumor microenvironment; TAMs, tumor-associated macrophages; IL-6, interleukin-6; IL-12, interleukin-12; TNF-alpha, tumor necrosis factor-alpha, TGFB1, transforming growth factor-beta-1, CCL2:C-C Motif Chemokine Ligand 2; ROS, reactive oxygen species.

Overall, the interaction between CSCs and the surrounding environmental cells is a complex and ever-evolving process. CSCs arise in “ecological” niches in the TME. These niches, establishing intense trafficking of factors, promote a stem-like and chemoresistant phenotype in the CSCs (10). In this scenario, emerging bioinformatics technologies, such as trajectory analysis and spatial transcriptomics, shed light on unresolved biological complexities. Particularly, these tools enable a deeper investigation of the crosstalk between CSCs and TAMs dissecting unrevealed aspects of their communication. Comprehending the intricate symbiotic relationships between CSCs and TAMs could provide valuable insights to identify an efficacious innovative therapeutic approach. An overview of CSC hallmarks and how these characteristics critically contribute to the complex interplay between CSCs and TME components is illustrated in **Figure 1**.

### From normal to CSCs endowed with tumor-initiation and metastatic potential

In normal adult tissues stem cells are undifferentiated cells that reside in a proper niche, where they are protected and can exert their functions. Stem cells show the ability of self-renewal and differentiation in adult cell tissue, maintaining tissue homeostasis. Stem cell niche can be identified in several tissues such as the crypts of the intestine, the bone marrow, the liver or lung tissues (11). After tissue injury, the niche transmits activation signals such as adhesion molecules, matrix proteins, oxygen, growth factors or cytokines to the stem cells for tissue regeneration. These signals are factors that allow cell-cell interactions between stem cells and neighboring differentiated cells (12).

In normal tissues stem cells remain in an undifferentiated state throughout adult life. Stem cells reach a first stage by becoming transient amplifying cells and highly proliferative cells then they asymmetrically divide and finally reach the last stage of differentiated cells, that leads them to build up and support tissues (13). In both stem and differentiated cells, the potential accumulation of intracellular pathways mutations can lead to a tumor-type phenotype (14). When a critical mutation threshold is reached, cells become CSCs, changing to a more aggressive behavior (15). CSCs, as normal stem cells, have the ability of self-renewal and differentiation, while they create a niche that proliferates independently of the surrounding tissue. These characteristics contribute to tumor initiation, growth and maintenance (13). Among the most deregulated intracellular pathways, wingless-related integration site (WNT)/beta-catenin, NOTCH and Sonic Hedgehog emerge, as they promote self-renewal and tissue morphogenesis (16). In addition, cellular growth, migration, differentiation and epithelial-mesenchymal transition (EMT) are regulated by phosphatidylinositol 3-kinase/AKT serine/threonine kinase 1/Phosphatase and tensin homolog (PI3K/AKT1/PTEN) axis, one of the majors signaling pathways in CSCs (17). TGF, SMAD, peroxisome proliferator-activated receptor (PPAR), mitogen-activated protein kinases (MAPK) and Janus kinase/signal transducers and activators of transcription (JAK-STAT) are often deregulated in CSCs (18). CSCs are not only involved in the process of tumor initiation, growth and maintenance, but also in metastasis (19). CSCs represent the most aggressive tumor subpopulation able to spread and form metastases even at distant sites. One of the key requisites for successful metastasis formation is stemness. Indeed, depletion of various stemness markers such as cluster of differentiation 44 (CD44) in breast CSCs (20) or octamer-binding transcription factor 4 (OCT4) and SRY-Box Transcription Factor 2 (SOX2) in colon CSCs, prevented tumor metastasis and tumor growth (21). Beyond stemness markers, several studies have been focused on the identification of cell-surface markers specifically expressed in the subpopulation of CSCs endowed with metastatic potential such as CD44v6, a CD44 variant isoform, in colon CSCs (22). A broad and extensive description of CSCs hallmarks and the methodologies used to characterize the CSC state is reported in (23). In the next paragraphs, we will briefly introduce how CSCs evade the immune system and resist conventional therapies.

### CSCs and immune evasion

Immunosurveillance is a set of immune-system related processes aimed at controlling the development of normal cells and detecting cancer cells. The innate and adaptive cells of the immune system respond to stress conditions, caused by tumor development, mainly by upregulating natural killer (NK) activator ligands and stimulating a more specific T lymphocyte response against cancer cells (24). NK cells are innate immune cells that recognize cells lacking major histocompatibility class I complex (MHC-I) and exert potent cytolytic activity releasing perforin and granzyme against transformed cells (24). NK cells mediate the tumor killing also triggering apoptotic pathways in tumor cells through the production of TNF-alpha or via direct cell–cell contact through activation of the Tumor necrosis factor (TNF)-related apoptosis-inducing ligand (TRAIL) and FAS ligand (FASL) pathways (24). Otherwise, T cells are the main component of the adaptive immunity that orchestrate a protective effector immune response, indeed, a high level of T cell infiltration in tumors is associated with a favorable prognosis in cancer patients (24). CD8+ T and CD4+ T helper 1 cells are the most prominent anti-tumor T cells, instead, through the exocytosis of perforin and granzyme containing granules, the former, and secretion of high amounts of proinflammatory cytokines, such as interleukin-2 (IL-2), TNF-alpha, and interferon-gamma (IFNG), the latter, promote T cell priming activation, cytotoxic T lymphocytes (CTL) cytotoxicity, but also, the anti-tumoral activity of macrophages and NK cells (24).

T and NK cells destructive effect on cancer cells is regulated even by TAMs, by increasing the number of active NKs, upregulating inhibitory T cell receptors programmed cell death protein 1 (PD-1) and Cytotoxic T lymphocyte associated protein 4 (CTLA-4), releasing factors such as TRAIL and inducing apoptosis in cancer cells (25, 26). However, during inflammation, TAMs can directly inhibit the proliferation of CD8+ T cell lymphocytes by regulating their metabolism or recruiting regulatory T cells (Tregs) (27). TAMs can also inhibit dendritic cell (DC) maturation and the secretion of IL-12 by DCs (28). TAMs and Tregs boost an immune-tolerant TME by secretion of molecules such as interleukin-10 (IL-10), TGFB1, and prostaglandins (28). Indeed, poor prognosis and reduced overall survival in oncological patients is correlated with high-grade TAMs (28). Tumor cells can evade the immune system by using different strategies like losing surface antigens that prevent recognition by cytotoxic T cells or downregulating cell surface NK activators, becoming invisible to detection by NK cells (28). However, the immune system can self-contribute to tumor development and progression, orchestrating an immunosuppressive inflammatory TME (24). This process is called “cancer immunoediting” and proceeds through three phases: elimination, equilibrium and escape (29). During the first phase the cytotoxic immune cells such as NK and CD8+ T cells kill transformed cells, although rare tumor subclones can survive (30, 31). These tumor subclones may enter the second phase where their growth is limited and stalled over time (30, 31). The steady pressure from the adaptive immune system and the genetic instability of cancer cells can make tumor subclones escaping immunosurveillance (30, 31). Cancer cells start proliferating unconditionally and adopt many features to escape from the immune system like downregulation of the antigen presentation machinery or inducting inhibitory immune checkpoint molecules (32). Moreover, cancer cells remodel the vasculature and extracellular matrix and supports cancer progression as well as therapy resistance (30, 31). This process can entail decreased IFN-gamma secretion by T cells, loss of antigen presentation and epigenetic changes (33).

Within the tumor CSCs control the immune system and regulate the composition of TME through the release of cytokines, chemokines, growth factors, metabolites and hormones playing an immunomodulatory role (34). CSCs develop different immunosuppressive strategies that promote tumor maintenance and growth. Downregulation of MHC-I complexes and activation of immune molecules such as cluster of differentiation 80 (CD80), human leukocyte antigen (HLA) and major Histocompatibility Complex Class I chain-related protein A/B (MICA/MICB), renders CSCs more resistant to cytotoxic effects exerted by CTL (35). Of note, the degree of tumor progression in the CSC niche has been attributed to a reduced CD8+ T cell infiltration and to an increase in TAMs (35). Moreover, CSCs interact through human leukocyte antigen G (HLA-G) with killer cell immunoglobulin like receptor, two Ig domains and long cytoplasmic tail 4 (KIR2DL4) and killer cell lectin like receptor C1 (KLRC1) to suppress NK activity (34). CSCs further drive recruitment and polarization Treg cells by secretion of factors like Chemokine (C-C motif) ligand 1 (CCL1), IL-2, interleukin-8 (IL-8), IL-10 and Transforming growth factor-beta-1 (TGFB1) (34). Moreover, Tregs produce TGFB1 and interleukin-17 (IL-17) to promote CSCs properties toward tumor progression and invasion (34). CSCs immune evasion properties are influenced by humoral factors: TGFB1, a cytokine that induces immune suppression, EMT and stemness; IL-6, secreted by TAMs, that induces and maintains CSCs, signal transducer and activator of transcription 3 (STAT3), a transcription factor required for the maintenance of pluripotency in stem cells or Chemokine (C-C motif) ligand 20 (CCL20) and its receptor that recruits Tregs to promote tumor progression enhanced by immune evasion (34). CTLA-4 and PD-1/programmed death-ligand 1 (PD-L1) represent two of the major immune checkpoints (34). Immunosuppressive myeloid cells, including macrophages and monocytic myeloid-derived suppressor cells (MDSCs) represent an additional layer of regulation of T cell activity and partially depend on secretion of factors like CSF1, CCL2, Chemokine (C-C motif) ligand 5 (CCL5), TGFB1 and prostaglandin E2 (PGE2), by CSCs (34). Collectively, all these interactions reshape the tumor microenvironment and create a habitat where immune cells support and are suppressed by CSCs (34).

### Therapy resistance in CSCs

Conventional therapies developed for cancer treatment are based on the following approaches such as chemotherapy, radiation therapy and surgical excision (36, 37). Chemotherapy is the most widely used and effective treatment for cancer; however, cancer cells as well as CSCs often elaborate simultaneous resistance to many drugs, even if they are structurally and functionally quite different (36, 37). This phenomenon is called multidrug resistance (MDR) or multifactorial pleiotropic drug resistance (36, 37). Many *in vivo* and *in vitro* studies demonstrated that administering chemotherapeutic drugs led to an enrichment in CSCs (36, 37). Drug resistance is caused by regular administration of chemotherapy drugs, that are dose- or time-dependent. The multiple mechanisms underlying MDR can be listed as follows: increased drug efflux and reduced drug uptake, efficient DNA repair mechanisms, reduced presence of reactive oxygen species (ROS), apoptosis evasion, hypoxia, vasculogenic mimicry (VM) activation, increased autophagy and decreased ferroptosis (38).

**Figure 2:**
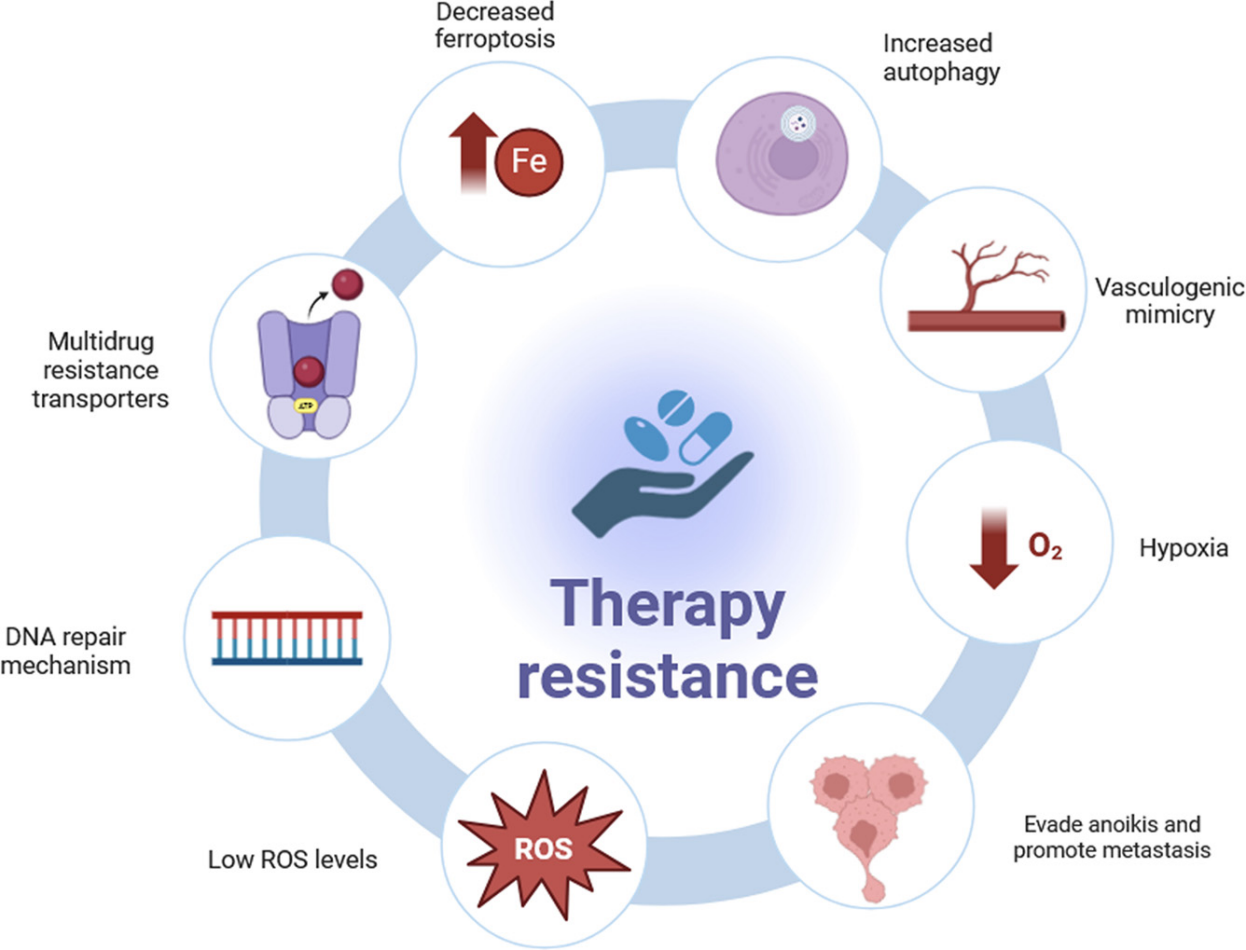
Mechanisms of therapy resistance in CSCs. Many CSCs strategies have been identified to resist to therapy: multidrug resistance transporters, efficient DNA repair mechanisms, lower Reactive Oxygen Species (ROS) levels, evading cell death or “anoikis” and promote metastasis, hypoxia, providing sufficient blood supply through vasculogenic mimicry (VM), increased autophagy and decreased ferroptosis.

Mechanisms responsible for therapy resistance in CSCs are summarized in **Figure 2**.

### Multidrug resistance transporters

Several studies demonstrated that many chemotherapeutic agents in clinical use are susceptible to ATP-binding cassette transporters-mediated efflux (ABC), such as microtubule-targeting, alkaloids, taxanes, topoisomerase inhibitors, DNA-damaging anthracyclines and tyrosine kinase inhibitors (38, 39). This subfamily of transporters is mainly localized in human tissues of the brain, lung, breast, kidneys, liver, ovaries, prostate, placenta and pancreas (40).

CSCs express higher levels of MDR transporters than cancer cells or healthy cells (41). ABCB1, ABCG2 and ABCB5 are overexpressed respectively in ovarian CSCs (41), breast CSCs (42) and malignant melanoma initiating cells (MMIC) (43). Inhibitors of the ABC transporters are currently used in clinical settings, although side-effects and high toxicity have been reported in patients (44).

### DNA repair mechanisms

Efficient DNA repair mechanisms in CSCs are thought to be a major contributing factor in counteracting treatment-induced DNA damage (45). Efficient DNA damage repair system and the CSC long permanence in a quiescent G0 phase greatly reduce potential exogenous and endogenous DNA damage that could occur during DNA replication (45). Evidence demonstrates that DNA damage response (DDR) sensor proteins are upregulated in CSCs rather than tumor bulk cancer cells in monolayer cultures, thus conferring radio and chemotherapy resistance (46). Enhanced expression of DNA polymerase nu (POLN) contributes to chemoresistance in ovarian stem cells (47). Thus, cytotoxicity by chemotherapeutic drugs or radiotherapy-induced can be attenuated in CSCs based on an efficient DNA damage repair system (26).

### ROS levels

CSCs show low intracellular levels of ROS, a group of highly reactive molecules, containing oxygen, that can promote DNA damage and influence the DDR machinery (48). Therefore, CSCs can dampen the entity of exogenous DNA damage induced by conventional therapy by expressing low levels of ROS, which production is mainly determined by the slow division rate of CSCs (48). Lower levels of ROS in CSCs result crucial in maintaining a stem cell-like phenotype, along with conferring resistance to radiation therapy and/or chemotherapy (49).

### Anoikis

The ability of CSCs to metastasize and reach other organs should be reduced as cells undergo programmed cell death or apoptosis, where they lose contact with their extracellular matrix or neighboring cells (“anoikis”) (50). However, CSCs were reported to be anoikis resistant (50). Indeed, CSCs endowed with metastatic potential evade anoikis mechanism, therefore surviving and promoting the formation of metastatic lesions at a distant site (51). Notably, co-culturing CSCs with non-CSCs conferred anoikis resistance to non-stem cells in breast cancer (51). CSC-like cells protected non-stem cells from anoikis and promoted tumor growth (51).

### Hypoxia

Oxygen is necessary for metabolism and cellular energy production. In many tumors, oxygen levels are usually between 0% and 2% compared to normal physiological levels that can reach up to 9% and therefore the high metabolic demand requires the activation of hypoxia-inducible factors (HIFs) (52, 53). HIFs are heterodimers consisting of two subunits a and b that can translocate in the nucleus and interact with specific sequences leading to activation or repression of gene expression (52, 53). There are three different genes encoding for HIF subunits: hypoxia inducible factor 1 subunit alpha (HIF1A), hypoxia inducible factor 2 subunit alpha (HIF2A), and hypoxia inducible factor 3 subunit alpha (HIF3A) (52, 53). All three heterodimerize with the hypoxia inducible factor 1 subunit beta (HIF1B) subunit and are subject to posttranslational regulation that is dependent on oxygen levels in the environment (52–54). HIF1A and HIF2A through the upregulation of regulators such as SOX2, Nanog homeobox (NANOG), OCT4, KLF Transcription Factor 4 (KLF4), and the transcription factor MYC proto-oncogene protein (MYC), have been shown to promote stemness and CSC phenotype (55). Upregulation of HIF-1 induces the expression of genes involved in angiogenesis, cell survival, and metabolism, conferring a selective advantage to CSCs (56). It has been demonstrated that breast cancer cells lines, MCF-7 and MDA-MB-231, display increased subpopulations of tumor cells with stem-like characteristics (56). Hypoxia is a hallmark of the CSCs environment that is essential for CSCs development, maintenance, tumor growth and resistance to therapy (57). Evidence suggests that the hypoxic niche in colon cancer protects CSCs from chemotherapy (58). Moreover, in ovarian cancer stem cell lines, SK-OV-3 and HO-8910, it has been demonstrated that chemotherapy treatment, under hypoxia conditions, induced CSC-like properties (59). The mechanisms through which hypoxia exerts its function are complex but can be summarized in shifting the metabolism toward aerobic glycolysis, reduced expression of pro-apoptotic factors, dysregulation of ROS and redox mechanisms, increasing genomic instability and aberrant cell cycling (48).

### Vasculogenic mimicry

Vascularization plays an important role during carcinogenesis and metastasis. VM can provide sufficient blood supply for tumor growth, independently of endothelial cells (60, 61). VM is a process of blood vessel formation that cancer cells and CSCs employ to increase the blood supply of angiogenesis (60, 61). It is a mimicry process whereby malignant cells mimic the function of endothelial cells to form blood vessels by reshaping the extracellular matrix (60, 61). CSC VM has been observed in many tumors such as breast cancer and melanoma (62). Evidence shows that vasculogenic mimicry is mostly present at the early stages of tumor development when blood supply is most needed, as the tumor grows where the vessels created by endothelial cells are established (63). Studies also show that the early stage of CSC serves as tumor vasculogenic stem/progenitor cells that can differentiate into tumor vasculogenic endothelial cells (64, 65). New vessel formation, and particularly VM, makes the eradication of the tumor even more complex and unsuccessful, giving the tumor the ability to metastasize (64, 65).

### Autophagy

Autophagy is a catabolic process that degrades and recycles cellular components and exhibits both protective and destructive roles in the TME under physiological stress conditions such as nutrient deprivation and hypoxia (66). The activation of autophagy may lead to an arrest of tumor development, but at the same time it can support CSC self-renewal and resistance to therapy (66). In CSCs, autophagy contributes to maintain self-renewal and proliferation properties, avoiding senescence (67). Evidence suggests that autophagy is involved in mechanisms that mediate resistance to therapy, in renal carcinoma and breast cancer (68, 69). Experiments carried out to inhibit autophagy have shown increasing sensitivity to radio- and chemotherapy in nasopharyngeal and breast CSCs, respectively (70, 71). In addition, the upregulation of signaling pathways mediating autophagy, such as SOX2- beta-catenin/BECLIN1, determines resistance to chemotherapy (72).

### Ferroptosis

Iron is an essential cofactor for several metabolic reactions and contributes to the formation of ROS (73). Ferroptosis can be defined as a form of iron-catalyzed necrosis and occurs through the intracellular accumulation of ROS, induced by lipid peroxidation (74). Current studies demonstrate that during tumor development the levels of iron and its transporters increase in CSCs compared to cancer cells (74). Although iron accumulation promotes ferroptosis, CSCs maintain a balance that prevents toxic lipid peroxidation (75). Chemotherapeutic drugs generate ROS that can induce oxidative damage and apoptosis (75). However, CSC ability to control ferroptosis reduces the harmful effect of ROS species conferring chemotherapeutic resistance (75). Inducing high levels of ferroptosis is indeed currently used as an innovative approach to revert chemotherapy resistance, specifically in the CSC population (76).

## TME as a key player in promoting CSC stemness and cancer development

**Figure 3:**
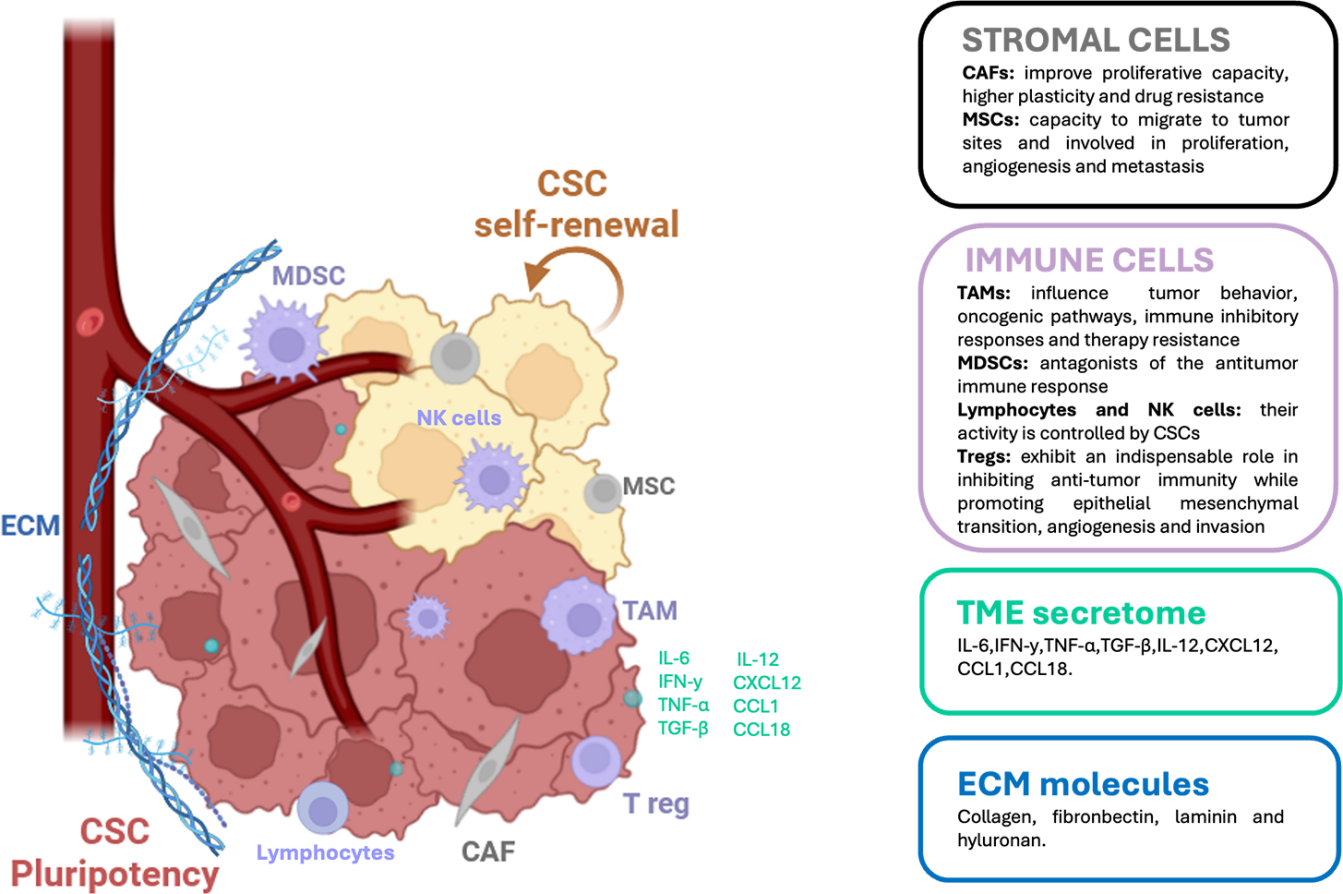
Tumor microenvironment (TME) key components. TME is a highly complex player composed of cellular components and non-cellular components, where cancer stem cells (CSCs) engage in communications with diverse immune cells, playing a critical role in cancer progression. CSCs have the ability of self-renewal (in yellow) and differentiation (in dark pink) in adult cell tissue, disrupting tissue homeostasis. Cellular components include: heterogenous cancer cells, diverse immune cells (e.g., T lymphocytes, regulatory T cell or Treg, tumor-associated macrophages or TAMs and myeloid-derived suppressor cells or MDSCs), stromal cells (e.g. cancer‐associated fibroblasts or CAFs and mesenchymal stromal cells or MSCs) and endothelial cells. Noncellular components include extracellular matrix (ECM) molecules (e.g., collagen, fibronectin, laminin and hyaluronan) biochemical and biophysical cues. Immune cells largely determine TME secretome composed of IL-6, IFN-gamma, TNF-alpha, TGFB1, IL-12, CXCL12, CCL1, CCL18 and several others. TME, tumor microenvironment; CSCs, cancer stem cells; TAMs, tumor associated macrophages; MDSCs, myeloid-derived suppressor cells; NK, natural killer; Treg, regulatory T cell; CAFs, cancer-associated fibroblasts; MSCs, mesenchymal stromal cells; ECM, extracellular matrix; IL-6, interleukin-6; IFNG, interferon-gamma; TNF-alpha, tumor necrosis factor-alpha; TGFB1, transforming growth factor-beta-1; IL-12, interleukin-12; CXCL12, C-X-C motif chemokine ligand 12; CCL1, chemokine (C-C motif) ligand 1; CCL18, C-C motif chemokine ligand 18.

Several studies have shown that various types of cells embedded in the TME contribute to maintain and sustain CSCs stemness properties. These findings prove that a crucial role in tumor progression is played by the specific TME surrounding tumor bulk cells and CSCs, which create the ideal conditions for tumor initiation. A detailed description of the key components present in the TME is reported in **Figure 3**.

TME includes various host healthy cells which enfold the tumor, and by producing cytokines and hormones they can promote its growth and behavior (77). As the core of the TME, tumor cells exploit cellular and non-cellular components for their own advantage by the installation of a complex signaling network (78). The host healthy cells, like fibroblasts or immune cells, as well as the extracellular matrix, undergo a tumor-mediated reprogramming able to convert the host cells into tumor associated ones such as cancer-associated fibroblasts (CAFs) and TAMs. Following the conversion, the tumor-associated cells start to sustain and promote tumor growth in different ways. Hereinafter, an overview of the main cells which compose TME and their contribution to tumor progression will be provided.

### Stromal cells and ECM

CAFs are highly heterogeneous stromal cells which represent the major modifiers of TME by the synthesis of soluble factors that promote tumor progression, stemness and angiogenesis in several cancers including prostate, gastric and non-small cell lung cancer (79–81). CAFs also contribute to tumor immune evasion both directly and indirectly. Different studies prove that CAFs are associated with T cells impairment, preventing their activation by secretion of C-X-C motif chemokine ligand 12 (CXCL12) and TGFB1 (82, 83). The primary role of CAFs is the establishment and apposition of the extracellular matrix (ECM) (84). ECM composes the scaffold for tissues and organs and facilitates cells crosstalk, both in healthy and malignant conditions. Jachetti et al. demonstrated that ECM proteins inhibit T cell proliferation and effector function (85). In addition, ECM can improve drug resistance by acting as a physical barrier. Besides, it has been shown that collagen, one of the most abundant proteins in ECM, can promotes stemness through the activation of an integrin/PI3K/AKT1/SNAIL signaling pathway (86).

Mesenchymal stromal cells (MSCs) are a substantial component of TME, recruited and re-educated by tumor cells in order to sustain tumorigenesis (87). Indeed, tumor associated-MSCs are crucial promoters of cancer hallmarks. It is shown that IL-6 produced by MSCs increases endothelin 1 (ET-1) expression in colorectal cancer (CRC) cells, resulting in the activation of AKT1 and ERK in endothelial cells which lead to tumor neo-angiogenesis enhancement (88). Several studies demonstrated that MSCs contribute also to tumor invasiveness and progression by regulating EMT regulators, like Twist, Snail and Zinc finger E-box binding homeobox 1 (ZEB1) (89–92). Finally, MSCs interact and suppress TME-embedded immune cells, either directly or through the release of factors like TGFB1, IL-2 and IL-10 (93) and, moreover, play a crucial role in enhancing stemness of cancer cells. Indeed, in physiological conditions, MSCs shape and support tissues and promote stemness features of the stem cell niches. Similarly, MSCs interact and promote CSC stemness in tumors via soluble factors (52).

### Immune cells

Although immune cells should prevent and resolve tumor progression, they act as promoters of cancer development under the pressure of TME signalosome (94). MDSCs are regulators of immune homeostasis (95). Cancer cells exploit MDSCs activity to escape immune surveillance, indeed MDSCs are commonly present in TME for their capability to facilitate tumor progression by establishing immune-suppressive conditions in different ways (96). ROS, IL-10 and TGFB1 produced by MDSCs negatively regulates CD8+ T cells activity against cancer cells (96). Moreover, MDCSs up-regulate PD-L1 expression, resulting in suppression of the immune response against tumors (97). MDSCs also regulate indirectly the immune response exacerbating TME by factors essential for T lymphocytes functions, such as L-arginine, which is crucial for T cells proliferation and activity (98, 99). MDSCs promote CSC stemness by miRNAs able to trigger CSCs stemness program (100).

Tregs are spontaneously attracted by immunosuppressive cytokines produced by tumor and tumor-associated cells (101). As well as MDSCs, Tregs promote tumors immune evasion by releasing cytokines able to suppress the activation of the immune response effectors (102, 103). Recent evidence suggests that Tregs are important regulators of CSCs stemness. Indeed, in several cancers, Tregs promote stemness-related pathways (104), facilitate EMT (105) and angiogenesis (101).

Tumor cells and TME not only re-educate and exploit MDSCs and Tregs but also induce depletion of tumor killing activity exerted by immune response effectors cells, NK cells and lymphocytic cells (106, 107). Although in the early stages of tumorigenesis NK cells are lethal for tumor cells, they slowly exhausted their killing function under the pressure of TME factors (108). Indeed, TGF-beta produced by CSCs, MDSCs and Tregs, impairs NK cells cytotoxicity, inhibits the release of IFNG and reduces the expression levels of killer cell lectin like receptor K1 (KLRK1) receptor in several tumors (109–111). Also, TME hypoxia conditions inhibit NK cells by downregulating expression of NKp46, NKp30, NKp44, KLRK1, perforin (PRF1), and granzyme B (GZMB) (112). Finally, lactate produced by tumor cells leads to the acidification of TME which induces apoptosis of NK cells (113). The same conditions which inhibit NK cells affect also lymphocytic cells activity, the most potent immune weapons against tumor cells (114, 115). Besides, downregulation of MHC-I, along with the up-regulation of immune checkpoints, (i.e. PD-L1) allows tumor cells to ensure themselves immune evasion (116, 117). Among the immune cells present in the TME, a focus on TAMs and their hallmarks will be provided in the next paragraphs.

Recently, CSCs-TME interplay gained interest in cancer research as a potential therapeutic target against tumors. TME promotes a stem-like state in CSCs supporting their self-renewal, survival, and therapeutic resistance through different molecular mechanisms (118). CAFs, the most represented cells in TME, release cytokines like IL-6, able to sustain the expression of stemness-related genes like SOX2, NANOG and OCT4 in CSCs (119). On the other hand, CSCs drive TME immunosuppressive polarization and persistence (35). CSCs can regulate immune system activity through the release of immunosuppressive secretome (i.e. IL-10, TGFB1) showing a more efficient capability to recruit immune cells with pro-tumoral activity (Tregs, MDSCs and especially TAMs) which sustain CSCs stemness by releasing factors like platelet-derived growth factor (PDGF), IL-8, CXCL12 (120, 121).

## The TAMS story

As tissue-resident immune cells, macrophages represent an anti-cancer first line of defense thanks to their capability to recognize and phagocyte malignant cells, but they are also the first allies of tumor initiation and development. After malignant transformation, TAMs are the result of the exploitation of macrophages plasticity (M1-M2 dichotomy), by cancer cells (122, 123). TAMs play a pivotal role in vascularization, inflammation, EMT and intravasation in different cancer models (124–127). This review aims to shed new light on the important role of macrophages in cancer development and the close link with TME modulation, the role of macrophages and monocytes, in relation with CSCs stemness and support.

## TAMs Hallmarks

The hallmarks of cancer, initially introduced by Hanahan and Weinberg (128) mirror the complex and fundamental biological mechanisms that drive cancer cells to malignancy. In this context, TAMs have emerged as crucial players, within the TME, in cancer progression showing ability in tumor growth and metastasis processes (129). Particularly, TAMs originate from circulating monocytes, in the bloodstream, that migrate to tumor sites where they become macrophages (130). Macrophages are characterized by a peculiar plastic phenotype and can differentiate in wide spectrum of subclasses finely driven by super-enhancers activity (131, 132). Usually, they are classified as M1 or M2, which display pro-inflammatory and immunosuppressive phenotypes respectively. In cancer contexts, TAMs mainly display an M2-like state, which is correlated to oncogenic features such as cancer cell proliferation, immunosuppression, chemoresistance, angiogenesis and metastasis (133). Overall, the acquisition of an M2-like state is critical to create a microenvironment that supports both the survival and progression of cancer cells (134). Moreover, an enrichment of TAMs infiltration, in the context of TME is linked to a worse prognosis in several cancers (135–137).

**Figure 4:**
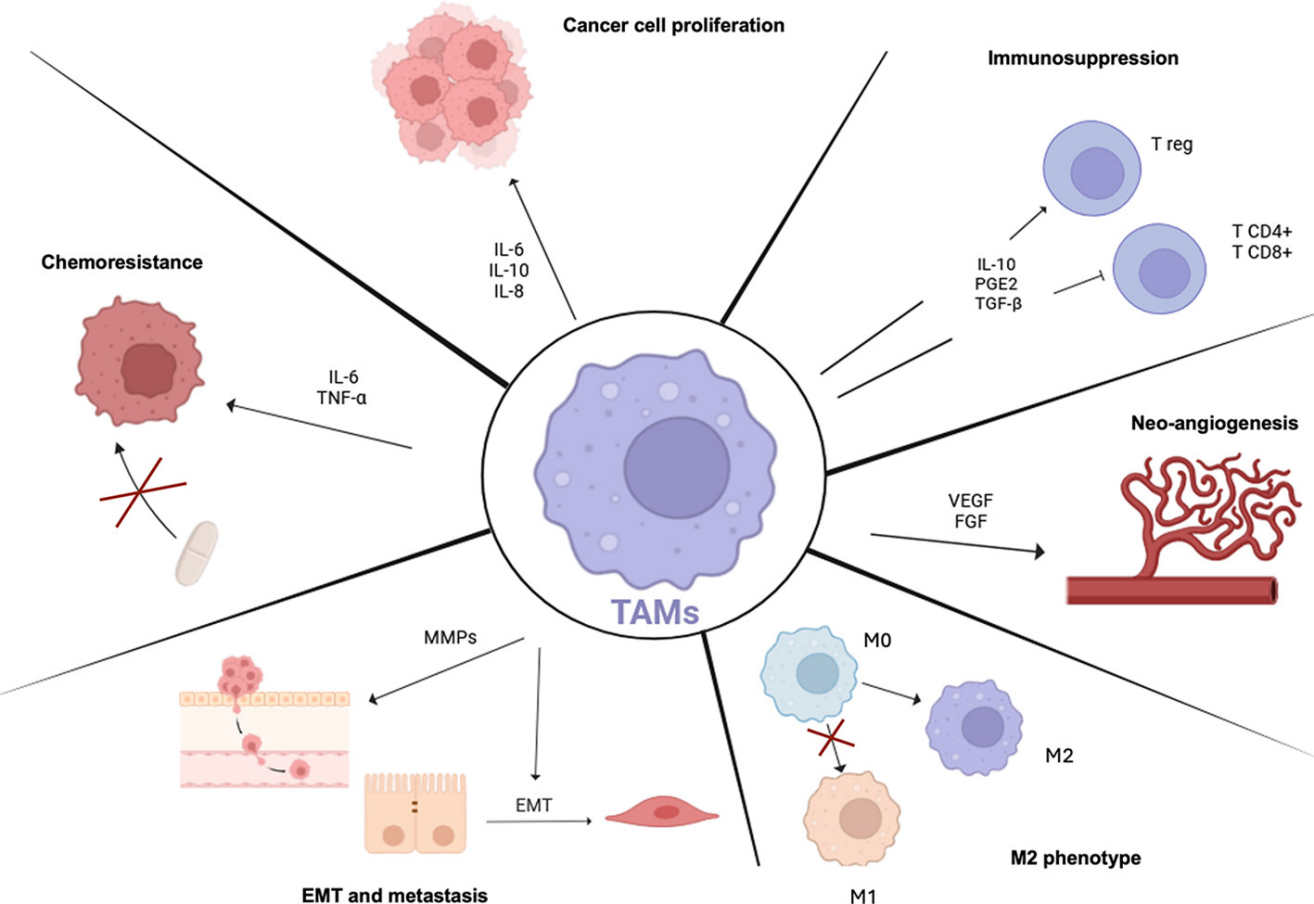
TAMs Hallmarks. Scheme showing TAMs properties in tumor progression. TAMs predominantly show an M2-like state which is mainly linked to pro-tumoral programs. TAMs are involved in many aspects of tumor cell biology such as T lymphocytes immunosuppression and increasing T reg recruitment, supporting tumor angiogenesis through pro-angiogenic factor production, inducing epithelial mesenchymal transition (EMT) and metastasis and promoting resistance to therapy activating pro-survival programs. TAMs, tumor-associated macrophages; IL-6, interleukin-6; IL-10, interleukin-10; IL-8, interleukin-8; Tregs, regulatory T cells; PGE2, prostaglandin E2; TGFB1, transforming growth factor-beta-1; VEGF, vascular endothelial growth factor; FGF, fibroblast growth factor; EMT, epithelial-mesenchymal transition; MMPs, matrix metalloproteinases; TNF-alpha, tumor necrosis factor-alpha.

The most significant TAMs hallmarks, which promote tumor progression are shown in **Figure 4** and detailed below.

### Cancer cell proliferation

A key hallmark of cancer is the ability to engage in an intricate communication with tumor cells and by activating proliferative signaling programs (138). TAMs positively support the cancer cell-cycle state by secreting various growth factors and cytokines. Among TAMs released factors, IL-6, IL-10 and IL-8 foster signaling pathways directly involved in stimulating cancer cell proliferation and tumor growth (139–142).

### Immunosuppression

TAMs predominantly display an immunosuppressive M2 state in TME (130). M2 TAMs unbalance the immune surveillance role of T cells and favor the promotion of cancer cells escape from the immune system (130). More in detail, TAMs produce a plethora of molecules, such as TGFB1, IL-10, and PGE2 that act on T cells, disrupting the anti-tumoral role both CD4+ and CD8+ subtypes, and increasing the recruitment of Tregs, that enhance the pro-tumoral immune depletion (130, 143).

### Chemoresistance

Chemoresistance is another cancer hallmark. TAMs play a crucial role in the acquisition of a cancer chemoresistant phenotype, through the secretion of inflammatory cytokines such as IL-6 and TNF-alpha which activate pro-survival programs (144, 145). Moreover, TAMs can enhance the efflux of chemotherapeutic drugs from cancer cells, reducing their therapeutic efficacy (146).

### Neo-angiogenesis

TAMs promote neo-angiogenesis, vital for both tumor growth and metastasis. Accordingly, TAMs release pro-angiogenic factors such as vascular endothelial growth factor (VEGF) and fibroblast growth factor (FGF), which drive the activation of new blood vessels formation signaling pathways (147, 148). The neo-angiogenesis not only is essential to feed cancer cells with nutrients and oxygen, but also it is critical for tumor mass growth and to guide metastatic spreading process (147).

### EMT and metastasis

The pro-invasiveness and pro-metastatic role of TAMs is well-documented in literature (5, 149). Accordingly, TAMs can induce EMT in tumor cells toward a more mesenchymal phenotype, enhancing their more malignant invasive phenotype (149). Furthermore, TAMs can secrete matrix metalloproteinases through which they digest the extracellular matrix components, allowing cancer cells to reach the surrounding tissues (150).

In summary, TAMs significantly impact multiple hallmarks of cancer. Through their roles in sustaining proliferative signaling, immunosuppression, chemoresistance angiogenesis and metastasis, TAMs represent a critical player in all stages of cancer progression, from early to late ones.

### CSCs and TAMs crosstalk and its impact on metastatic niche

One of the major drawbacks in counteracting cancer spread and resistance consists of the capacity of CSCs to migrate into secondary sites and avoid immune surveillance (2). Given their plastic behavior, their self-renewal capacity and treatment resistance, CSCs can foster metastasis formation from the primary tumor environment by disseminating into further districts, establishing the metastatic niche (151). In this context, a model that explains the architecture of the niche has been proposed by Lyden et al., in which the CSCs by migrating, reach a permissive and suitable microenvironment, the pre-metastatic niche, and by becoming disseminated tumor cells (DTCs), they can colonize and proliferate (152) through direct competition with normal stem cells for the niche occupation and establishment (153).

The metastatic niche characteristics vary depending on the specific components considered: the interacting cell types, ECM proteins, survival and self-renewal signals, but mostly the secondary site locations, that can either sustain and foster the metastatic niche, or set a hostile environment for the DTCs (154). The DTCs have to face several issues when colonizing a secondary site, including the lack of growth and extracellular matrix remodeling factors, that can hamper their survival and proliferation, thus adjusting into the new niche and metastasize (155). The disseminated cells will shape their surroundings to build a supportive metastatic niche and exploit the functions of both CSCs and metastatic stromal cells (2). However, studies analyzing human colorectal cancer samples displayed that metastatic occurrence arises from primary tumor cells, that are resistant to chemotherapy and might stay quiescent for a prolonged time (156).

Notwithstanding, little evidence investigating the genetic profiling of the tissue-derived and metastatic CSCs emerged, in the consideration of the metastasis signature mutations occurrence at the level of the primary tumor. This process can represent the first tool of selection in the CSCs population to direct a pool toward migration and extravasation into secondary sites (151). With this premises, the most accredited option relies on the fact that metastasis-driving alterations are present within the heterogeneous CSCs profile, and their expression selects the DTCs that will acquire a plastic and resistant profile (157).

Nevertheless, further evaluations need to be carried out, especially in the context of EMT pathways, and stem-like features involved in both the primary tumoral site and in the metastatic environment, highlighting similarities and differences among CSCs and DTCs (158).

Historically, the metastatic niche has been described as a cell-enriched environment constituted mostly by immune and stromal cells which secrete proteins and factors that sustain growth and self-renewal of CSCs, that consequentially stimulate the activation of angiogenic pathways aimed to the promotion of tumor invasion and metastatization (154, 159).

CSCs form the primary tumor can favor the diffusion of pro-tumorigenic and proangiogenic factors such as VEGF-A, TGFB1, TNF-alpha and lysyl oxidase (LOX) that induce the expression of S100A (a Ca2+ binding protein involved in endothelial remodeling) in the metastatic area (2). In the metastatic site the vasculature system boosts the recruitment of metastatic cancer stem cells (MetCSCs) by producing fibronectin and vascular endothelial cell adhesion molecule (VCAM). Consistently, it has been observed that the CCL2-CCR2 (C-C chemokine receptor type 2) axis promotes the establishment of inflammatory monocytes to the metastatic site, where they will transition into metastatic-associated macrophages (MAMs) and will enhance the extravasation and survival of metastasis-resident cancer cells (160).

Another important lead of metastasis formation is depicted by TAMs. Principally, TAMs promote tumor cell invasion and dissemination, and through their ability to release cytokines and factors that support growth and ECM-shaping (MMP-2, MMP-9), milk fat globule-EGF factor 8 (MFGE8), IL-6 are correlated with tumor progression and metastasis (161). TAMs derive from circulating Ly6C+CCR2+ inflammatory monocytes that are produced by hematopoietic stem cells (HSCs) in the bone marrow, that, when interacting with tumor tissue, are addressed toward a more cancerous-like profile (161, 162).

TAMs and CSCs crosstalk has been widely described in the last years, investigating whether their interaction may be direct or indirect, and which may be the effects on CSCs in the primary tumor, including chemoresistance, differentiation and proliferation (163). TAMs are essential in supporting metastasis establishment once CSCs migration has occurred. More specifically, studies conducted on lung and liver metastatic murine models, showed how inhibiting TAM recruitment in metastatic niches resulted in a reduced burden, indicating its paramount role in the onset and maintenance of metastasis by supporting both extravasation and intravasation in secondary sites of CSCs (164, 165).

One accredited metastasis hypothesis linking the role of TAMs in facilitating CSCs metastatization relies on the ability of metastatic cells to occupy niches in which are present CSCs (166). More in detail, it is thought that TAMs and CSCs derive from cell hybrids and set metastasis in further sites (166). The theory was proposed by John Pawelek in 2006, and he explained that myeloid and tumoral cells could perform a genomic hybridization (167). TAMs due to their migratory ability and the tissue-repair feature could transport the CSCs spheroids through either bloodstream or lymphatic circulation, and permit a favorable environment for metastatic initiation (168). Within the metastatic microenvironment, TAMs play a crucial role in shaping the behavior of CSCs, especially regarding tumor advancement and the colonization of cancer cells at secondary sites (169). A crucial aspect of TAMs is their role in promoting EMT, which is a vital process in the morphological alterations of cancer cells and contributes to the enhancement of their malignant traits (170). In triple-negative breast cancer, CCL2 secreted by TAMs activates AKT signaling pathways, resulting in heightened beta-catenin activity in CSCs (171). This pathway is essential for facilitating EMT and sustaining the properties of CSCs within the TME (172). In oral squamous cell carcinoma (OSCC), high levels of TAM-derived IL-6, promote EMT and enhance the expression of genes associated with stemness, via the IL-6/STAT3/thrombospondin 1 (THBS1) signaling pathway (173).

To sustain CSCs in pancreatic ductal adenocarcinoma (PDAC), TAMs utilize a critical mechanism involving the interferon-stimulated gene 15 (ISG15) signaling pathway (174). By releasing the ISG15, TAMs enhance the self-renewal, invasive potential and tumorigenic capabilities of CSCs (175). Among the several ways in which TAMs support CSCs behavior, the creation of an immunosuppressive microenvironment exerts a key function. Within the TME, TAMs predominantly display a M2 phenotype, which is known for its role in promoting immunosuppression (176). This phenotype fosters a protective microenvironment that shields CSCs from immune system attacks. By releasing immunosuppressive cytokines like IL-10 and TGFB1, TAMs effectively suppress the function of cytotoxic T cells and other immune cells, allowing CSCs to remain undetected and avoid destruction (177). TAMs influence the growth of CSCs through both direct contact and secretory mechanisms. In highly metastatic breast cancer, CSCs express hyaluronan synthase 2 (HAS2), which is crucial for creating a pro-metastatic microenvironment (177). This expression facilitates interactions between CSCs and TAMs, leading to the secretion of platelet-derived growth factor-B subunits (PDGFB) by TAMs (177).

PDGFB subsequently stimulates bone stromal cells to secrete fibroblast growth factors 7 and 9 (FGF7 and FGF9), which support CSC proliferation and survival (178). Moreover, in breast cancer, the EMT enhances the expression of cluster of differentiation 90 (CD90) and ephrin type-a receptor 4 (EPHA4), facilitating direct physical interactions between CSCs and TAMs through the binding with their respective receptors. When the EPHA4 receptor on carcinoma cells is activated, it triggers the sarcoma SRC proto-oncogene, non-receptor tyrosine kinase (SRC) and nuclear factor- kappa B (NF-kappa-B) signaling pathways. This activation leads to NF-kappa-B in CSCs induction of the secretion of various cytokines that help maintaining the stem cell state (179).

By preserving the stem-like properties of CSCs and boosting their migratory and invasive abilities, TAMs facilitate the detachment of CSCs from the primary tumor, enabling the formation of secondary tumors in distant organs (149).

Once malignant cells escape from the primary tumor, they intravasate and disseminate through the lymphatic and/or circulatory system, eventually establishing secondary tumors at distant sites. Research into lung metastasis reveals that when tumor cells reach their target location, they form micro-clots in conjunction with platelets, resulting in their entrapment within the blood vessels of the target tissue (180). Once arrested, the tumor cells secrete CCL2, which creates a gradient that attracts Ly6C monocytes (181). These recruited monocytes undergo differentiation into MAMs, which play a pivotal role in facilitating the extravasation of tumor cells by releasing VEGF, a factor known to enhance vascular permeability (182). Under the influence of CSF1, the primary lineage regulator for most macrophage populations, MAMs support the survival of tumor cells and contribute to their sustained growth through processes related to angiogenesis (183).

Recent studies conducted on CRC evidenced the interaction of CRC cells and TAM. Of note, a paramount interaction between CRC cells and M2 macrophages in the promotion of colorectal liver metastasis (CRLM) emerged (184). To date, CRLM is mediated by interactions between tumor cells and the TME in the liver and is considered one of the most common secondary liver cancers (185). Nevertheless, the mechanisms involved in the cancer cell-derived activation of M2 macrophages need further investigations in both CRC and CRLM. Notably, exosomes derived from tumors can polarize macrophages toward a M2 cellular profile, which in turn promotes metastasis (186, 187). Zhao et al, demonstrated that exosomes derived from CRC cells displayed a role in inducing M2 polarization through the secretion of microRNA-934 (miR-934) and the downregulation of PTEN expression, and activation of PI3K/AKT signaling cascade. Finally, miR-394 activated polarized M2 macrophages which promoted CRLM through C-X-C motif chemokine ligand 5 and 13 (CXCL5)/(CXCL13)/NF-kappa-B/p65/miR-394 positive feedback mechanism (188).

Another study (189) conducted on glioblastoma investigated the role of glioblastoma stem cells (GSCs) and TAMs in tumor progression and metastatic potential. The authors screened GSCs factors that could polarize macrophages, and they evaluated a potential group of proteins produced by GSCs with the ability of behaving as TAMs chemoattractant (189). Periostin (POSTN) emerged as a valuable factor expressed by the stem cells (189). It plays a role in the PI3K/AKT and WNT signaling pathways, which are involved in tumorigenesis (190, 191). In particular, evidence highlighted that CSCs profited from the POSTN-induced WNT augmented signaling, supporting a favorable metastatic colonization in breast cancer setting (192). Additionally, when silencing POSTN, TAM density was sensibly reduced, thus reinforcing the idea that GSCs can recruit TAMs and foster tumor growth by secreting POSTN. Consistently, GSCs established in the tumoral area, where they exploited the surrounding microenvironment by attracting TAMs from the peripheral circulation to set a more beneficial space for the reciprocal survival and growth of the resident populations and enhancing the metastatic CSCs potential. These observations need further investigation and open new scenarios regarding the involvement of TAMs, the role of CSCs, and their complex interplay in affecting the metastatic niche.

### TAMs influence on tumor behavior, oncogenic pathways, immune inhibitory responses and therapy resistance

TAMs represent a cellular immune system subpopulation directly involved in the tumor formation and progression through the activation of several pro-tumoral signaling pathways within the CSCs, thus providing the creation of a tumor niche necessary for CSCs survival and expansion. Cellular matrix elements represent critical components for the tumor niche structure maintenance which help the direct crosstalk between CSCs and the surrounding cells, including TAMs. The intricate bi-directional communication between TAMs and CSCs is increasingly recognized as a critical factor in tumor biology. This interaction is underscored by a growing list of factors, ligands/receptors, shown in **Table 1**, derived from both TAMs and CSCs that are implicated in the mutual co-dependent maintenance of CSC stemness and the supportive actions of TAMs. The complex network of signaling molecules and pathways involved in this crosstalk not only influences tumor progression but also impacts therapeutic resistance, making it a focal point for cancer research.

**Table 1:** CSCs-TAMs crosstalk and signaling pathways in cancers.

Target molecule	Target cell	Mechanism of action	Promoting characteristics	CSCs-TAMs interaction	Cancer subtype	Reference
CCL2	TAMs	Activates AKT signaling pathways in CSCs	Enhances beta-catenin activity, facilitates EMT, sustains CSC properties	TAM-mediated CSC support	TNBC	(172)
IL-6	TAMs	Promotes EMT via IL-6/STAT3/THBS1 signaling pathway	Enhances stemness gene expression	TAM-mediated CSC support	OSCC	(173)
ISG15	TAMs	It is expressed and secreted in response to IFN-beta produced by PDAC cells and acts on PDAC CSCs	Enhances self-renewal, invasive potential, and tumorigenic capabilities of CSCs	TAM-mediated CSC support	PDAC	(174, 175)
PDGFB	TAMs	Stimulates bone stromal cells to secrete FGF7 and FGF9	Supports CSC proliferation and survival	TAM-mediated CSC support	Breast cancer	(178)
EPHA4 and CD90	CSCs	Facilitate direct physical interactions between TAMs and CSCs	Maintain stem-like properties, boosts migratory and invasive abilities of CSCs	specific CSC-TAM signaling pathways	Breast cancer	(196, 282)
POSTN	CSCs	Involved in PI3K/AKT and WNT signaling pathways	Supports metastatic colonization	specific CSC-TAM signaling pathways	Breast cancer	(192)
HAS2	CSCs	Promotes hyaluronic acid synthesis, interacting with TAMs; Stimulates TAM release of PDGFB	Induces CSC self-renewal	specific CSC-TAM signaling pathways	Breast cancer	(177)
hCAP-18/LL-37	TAMs	Anchors FPR2 and P2X7R on pancreatic cancer cells	Activates stemness genes driving self-renewal, invasion, tumorigenicity	TAM-mediated CSC support	PDAC	(198)
TGFB1	TAMs	Induces EMT program activation	Enhances the CSC-like phenotype	TAM-mediated CSC support	HCC	(120)
CCL18	TAMs	Regulates metastasis through EMT program activation	Supports stemness	TAM-mediated CSC support	SCCHN	(201)
miR-221-3p	TAMs	Reduces transcription of ADAMTS6, induces AKT signaling	Boosts EMT program, promotes acquisition of a CSC-like phenotype	TAM-mediated CSC support	EOC	(203, 204)
MGF-E8 and IL-6	TAMs	Induces STAT3 and SHH signaling pathways in NSCLC stem cells	Leads to chemoresistance	TAM-mediated CSC support	NSCLC	(213)

Table summarizing CSC-TAM crosstalk and signaling pathways in cancers.

TAMs, tumor-associated macrophages; CSCs, cancer stem cells; TNBC, Triple-negative breast cancer; OSCC, Oral squamous cell carcinoma; PDAC, Pancreatic ductal adenocarcinoma; HCC, Hepatocellular carcinoma; SCCHN, Squamous cell carcinoma of head and neck; EOC, Epithelial ovarian carcinoma; NSCLC, Non-small cell lung cancer; CCL2, chemokine (C-C motif) ligand; IL-6, Interleukin 6; ISG15, Interferon-stimulated Gene 15; PDGFB, Plateled-derived growth factor B subunits; EPHA4, Ephrin type-A receptor 4; CD90, Cluster of Differentiation 90; POSTN, Periostin; HAS2, Enzyme hyaluronan synthase 2; hCAP-18/LL-37, Immunomodulatory cationic antimicrobial peptide 18/LL-37; TGFB1, Transforming growth factor-beta-1; CCL18, chemokine (C-C motif) ligand 18; miR-221-3p, MicroRNA-221-3p; MGF-E8, Milk fat globule epidermal growth factor 8; AKT1, AKT serine/threonine kinase 1; EMT, epithelial-mesenchymal transition; STAT3, signal transducer and activator of transcription 3; THBS1, thrombospondin 1; FGF7, fibroblast growth factor 7; FGF9, fibroblast growth factor 9; PI3K, phosphatidylinositol 3-kinase; FPR2, formyl peptide receptor 2; P2X7R, P2X purinoceptor 7 receptor; ADAMTS6, ADAM metallopeptidase with thrombospondin type 1 motif 6; SHH, Sonic hedgehog; WNT, wingless-related integration site.

In breast cancer stem cells (BCSCs) the overexpression of the HAS2 is implicated in the new synthesis of hyaluronic acid, a major polysaccharide component of the ECM which drives the physical interaction to TAMs, via CD44 receptor expressed on their surface. The hyaluronic acid/CD44 interaction stimulates TAMs to release the growth factor PDGFB, which induces CSC self-renewal (178, 193). In addition, TAMs/CSCs *in vitro* co-culture confirmed the oncogenic role of hyaluronic acid-expressing CSCs/CD44-TAMs interaction in the activation of different signaling pathways such as PI3K–Eukaryotic Translation Initiation Factor 4E Binding Protein 1 (EIF4EBP1)–SOX2, implicated in CSCs pool maintenance (194, 195). Interestingly, it has been found that BCSCs cooperate directly with TAMs through cluster of differentiation 11b (CD11b) and CD90 binding. This anchoring stimulates EPHA4 receptor-mediated induction of both the NF-kappa-B and SRC signaling pathways ensuring CSCs pool stemness state (196). Similarly, in a triple negative breast cancer (TNBC) model, the butyrophilin subfamily member A3 (BTN3A3) receptor enhances cancer stemness markers (i.e. NANOG, OCT4, SOX2) via juxtacrine interaction with its ligand, liver and lymph node sinusoidal endothelial cell C-type lectin (LSECtin), a transmembrane protein expressed on TAMs surface (197). Furthermore, CSCs engage a juxtacrine signaling pathway with the TAMs via GPI-anchored protein CD90/CD11b. Specifically, CSCs express the membrane GPI-anchored protein CD90 and EPHA4. Mechanistically, CD90 creates a bridge to bind the integrin CD11b on TAM surface, whereas the receptor EPHA4 interacts with its ligand, Ephrin, expressed by TAMs, inducing the expression of both SRC and NF-kappa-B driving tumor progression and metastatic dissemination (196). In pancreatic cancer, immunomodulatory cationic antimicrobial peptide 18/LL-37 (hCAP-18/LL-37) on TAM, anchors the formyl peptide receptor 2 (FPR2) and the P2X purinoceptor 7 receptor (P2X7R) expressed on pancreatic cancer cells, which lead to the activation of stemness genes (i.e. KLF4, SOX2, OCT3/4 and NANOG) driving CSC self-renewal, invasion, tumorigenicity (198).

Different studies showed that an indirect paracrine interaction between TAMs and CSCs, driven by a plethora of inflammatory molecules including cytokines, chemokines, growth factors, was also crucial in the determination of CSCs fate and behavior. Particularly, IL-6 is one of the most representative pro-inflammatory cytokines in the context of TME. It is critically upregulated in many tumors, underlying the strong correlation between inflammatory stimuli and tumor progression by affecting multiple cancer signaling pathways (199). IL-6 derived from TAMs induces the proliferation of CD44+ Human Hepatocellular Carcinoma Stem Cells (HHCSCs) via STAT3 pathway induction (139). In addition, TAMs secrete high levels of IL-6 increasing stemness markers (i.e. SOX2, OCT3/4 and NANOG) and consequently CSCs expansion in breast cancer cells via STAT3 pathway supporting tumor cells migration and angiogenesis (125).

Paracrine communication mechanisms between TAM and CSCs are driven by several molecules. TAMs can enhance the CSC-like phenotype via TGFB1, which induces EMT program activation in a hepatocellular carcinoma (HCC) (120). Similarly, TAMs induce stemness, EMT and chemoresistance in HCC by realizing TNF-alpha via the WNT/β-catenin axis (200). It has been discovered that TAMs can produce Chemokine (C-C motif) ligand 18 (CCL18). In squamous cell carcinoma of the head and neck model (SCCHN), CCL18 produced by TAMs regulates metastasis through the activation of EMT program and cancer stemness (201). TAMs releasing CCL2 is correlated with worse prognosis in breast cancer. Particularly, TAM-produced CCL2 in the context of breast cancer microenvironment activates AKT/beta-catenin signaling resulting in EMT and CSC properties in TNBC (172).

Exosomes derived from TAMs have shown unrevealed aspects about the role of TAMs in the support of cancer progression. Specifically, it has been found that annexin A3 (ANXA3)-loaded exosomes derived from TAMs impaired ferroptosis process in laryngeal cancer cells supporting lymphatic metastasis. More in detail, ANXA3 in exosomes regulates negatively the ubiquitination of activating transcription factor 2 (ATF2), a transcription factor that induces ChaC Glutathione Specific Gamma-Glutamylcyclotransferase 1 (CHAC1) expression, thus blocking ferroptosis in lung squamous cell carcinoma (LSCC) cells (202). Moreover, CD163+ TAMs release exosomes that are absorbed by epithelial ovarian cancer cells (EOCCs) (203, 204).

During the tumor progression, TAMs can create an immunosuppressive TME facilitating the immune escape of CSCs. The creation of an immunosuppressive milieu depends on a fine balance between the inhibition of pro-inflammatory immune cells and the activation of immunosuppressive TAMs-dependent counterparts. Accordingly, TAMs promote the upregulation of cluster of differentiation 47 (CD47) ligand on different cancers stem cells (including pancreatic, HCC and leukemia), which interacts to signal-regulatory protein alpha (SIRPA) on immune cells inhibiting phagocytic process (205–207). Parallelly, TAMs can also inhibit the adaptive immune system. Particularly, TAMs boost both inhibitor immune checkpoints expression PD-1 and its ligand PD-L1 in T cells and CSCs, respectively (208). The concomitant expression of PD-L1 and PD-1 impedes the cytotoxicity in T-cells (208).

Overall, some evidence showed how TAM-derived factors and TAM-CSCs physical interactions drive the activation of a great number of pathways in CSCs that are responsible of the maintenance of stemness in different cancer histotypes. These stemness-related hallmark pathways include Sonic hedgehog (SHH), STAT3, NOTCH, PI3K/AKT, WNT/beta-catenin, and NANOG (18). Particularly, TAMs induce STAT3 pathway regulating the expression of stemness genes, via NF-kappa-B activation, in CSCs in different malignancies including breast cancer, liver cancer, prostate cancer, pancreatic cancer and colon cancer (139, 209–213). TAMs activate WNT/beta-catenin and SHH pathways, in CSCs, by leading transcriptional activation of stemness related genes in liver cancer, prostate cancer and lymphoma after secreting TNF-alpha, CCL5, pleiotrophin respectively (200, 211, 214). Furthermore, TAMs support cancer stemness through the direct activation of SHH pathway or through the induction of stemness-related alternative pathways (196, 213, 215–217). Specifically, TAMs sustain stemness via direct activation of SHH pathway in colon cancer (213), meanwhile SHH alternative signaling pathways are TAM-induced in pancreatic cancer (TGFB1/SMAD2/SMAD3/NANOG pathway) (215), in liver cancer (via the NOTCH pathway) (216), breast cancer (via the SRC Proto-Oncogene, Non-Receptor Tyrosine Kinase (SRC) pathway) (196), and in glioma via extracellular regulated kinase 1/2 (ERK1/2) pathway (217).

Innovative studies indicate that the complex communication between CSCs and TAMs has a critical pivotal role in the acquisition of a chemoresistant phenotype refractory to anticancer therapies. In OSCC TAMs influence positively the formation of CSC-like cells, via the induction of stemness markers of the SOX2, OCT4, and NANOG genes, leading to a strong reduction of the percentage of apoptosis in OSCC, supporting cell migration and chemoresistance to vincristine (218). Similarly, TAMs release Pleiotrophin (PTN), which interacts with the protein tyrosine phosphatase receptor type Z1 (PTPRZ1) receptor on the surface of CSCs, in OSCC model. The ligand/receptor interaction activates the FYN proto-oncogene (FYN)-AKT pathway, sustaining both the expression of stemness characteristics in CSCs and chemoresistance in tumor cells (219). Furthermore, MFGE8 in cooperation with IL-6, from TAMs induces both STAT3 and SHH signaling pathways in non-small cell lung cancer stem cells (NSCLCCSCs) leading to chemoresistance (213). Despite a growing body of research that has elucidated various molecular mechanisms underlying the interactions between TAMs and CSCs, significant gaps in our understanding remain. The intricate crosstalk between these two cellular populations is a complex phenomenon that has not yet been fully characterized.

**Figure 5:**
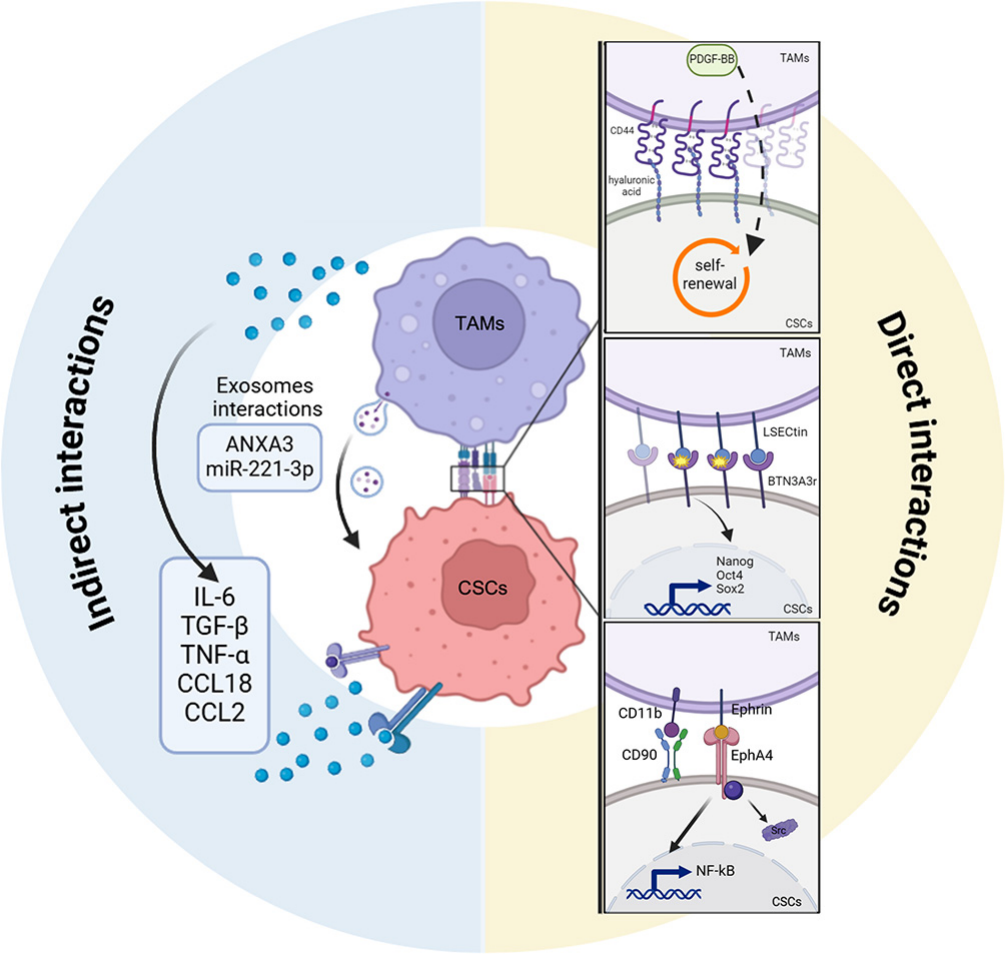
Indirect and direct interactions between CSCs and TAMs. Scheme showing the indirect (*left*) and the direct (*right*) mechanisms of crosstalk between tumor associated macrophages (TAMs) and cancer stem cells (CSCs). CSCs directly regulate TAMs activity to improve their own stemness conditions through different ligand/receptor interactions (hylaronic acids/CD44, BTN3A3r/LSECtin, CD11b/CD90, Ephrin/EPHA4. TAMs secretome including cytokines (IL-6, TGF- β, TNF-alpha, CCL18, CCL2) or exosomes cargo (ANXA3, microRNA-221-3p or miR-221-3p) promotes, indirectly, CSCs stem-like state by activating CSCs stemness programs. TAMs, tumor-associated macrophages; CSCs, cancer stem cells; CD44, cluster of differentiation 44; BTN3A3, butyrophilin subfamily member A3; LSECtin, liver and lymph node sinusoidal endothelial cell C-type lectin; OCT4, octamer-binding transcription factor 4; SOX2, SRY-Box transcription factor 2; CD90, cluster of differentiation 90; CD11b, cluster of differentiation 11b; EPHA4, ephrin type-a receptor 4; Src SRC Proto-Oncogene, Non-Receptor Tyrosine Kinase; NF-kappa-B, nuclear factor-kappa B; IL-6, interleukin 6; TGFB1, transforming growth factor-beta-1; TNF-alpha, tumor necrosis factor-alpha; CCL18, chemokine (C-C motif) ligand 18; CCL2, chemokine (C-C motif) ligand 2; ANXA3, annexin A3.

An overview of the most significant mechanisms of indirect and direct interaction between TAMs and CSCs are shown in **Figure 5**.

## Therapeutic strategies targeting the interactions between CSCS and TAMS to improve cancer treatment outcomes

The innovative targeting of the crosstalk between TAMs and CSCs represents a promising frontier in cancer therapy, although several strategies have been already developed to specifically target the CSC subpopulation including differentiative agents, chimeric antigen receptor T cell (CAR-T) therapy, natural compounds and epigenetic inhibitors (56, 220–225). This interaction is crucial as TAMs can enhance the stemness and survival of CSCs, contributing to tumor progression and resistance to conventional treatments (163). Nowadays the aim is to disrupt this communication, for the development of more effective therapeutic strategies that could potentially improve cancer patient prognosis. A therapeutic strategy could be represented by the disruption of CSC-TAM communication centers by blocking soluble factors that reciprocally support each cell type.

IL-6 is an important regulator in paracrine communication between TAMs and CSCs (125, 139). The IL-6 downstream pathway can be unpaired by both anti-IL-6, interleukin-6 receptor (IL-6R) antibodies and by STAT3 inhibitor pathway. Inhibitors against TGF-beta pathway are crucial to target CSCs (226, 227). Additionally, it has been discovered that IL-6 inhibition can impair MFGE8 functionality, which sustains CSC phenotype and cancer chemoresistance (213). Notably, the anti-IL-6R, tocilizumab, has been approved by the FDA for treating rheumatoid arthritis (228). It is currently in phase II clinical study for the treatment of unresectable late-stage melanoma in combination with the anti PD-1 and anti CTLA-4 immune checkpoint inhibitors nivolumab and ipilimumab (NCT03999749) (229). IL-8 is another important TAM-secreted regulator in cancer stemness (230). Reparixin is an anti-IL-8 receptor (IL-8R), known as CXCR1, that reduces CSC population in breast cancer setting (231). Phase I clinical trial study NCT02001974 showed that Reparixin provides a synergistic effect in combination with paclitaxel (231). The inhibition of the glioblastoma multiforme (GBM) CSC-released POSTN has shown a significant reduction in TAMs recruitment in pre-clinical glioblastoma model xenografts (189). In addition, Huang et al. demonstrated that TAMs-secreted CCL5 inhibition could impair stemness and metastasis formation in in pre-clinical prostate model xenografts (211). An alternative targeting strategy is to re-educate the biological role of TAMs toward an anti-tumor phenotype. Specifically, it has been demonstrated that dasatinib inhibitors, directed against SRC, drive the reprogramming from TAMs to M1 anti-tumor macrophages affecting the SRC/cluster of differentiation 155 (CD155)/macrophage inhibitory factor (MIF) signaling (232). This leads to downregulation of stemness markers, NOTCH1 and beta-catenin in cisplatin-resistant lung cancer cells (232).

The reactivation of phagocytic activity in anti-tumoral macrophages toward dead tumor cells represents a really important resource for obtaining cancer cells antigens to boost T cell-mediated immune responses. Accordingly, macrophage phagocytosis can be restored via anti- CD47 administration in immunodeficient pre-clinical xenograft models (233–236). Particularly, anti-CD47 antibodies are currently being designed in clinical trials (NCT02216409, NCT02367196) to overcome the phagocytosis-driven CD47+ TAMs/SIRPA+ CSCs inhibition with promising results (235, 237).

Interestingly, pre-clinical models showed a strong synergism between anti-CD47 and chemotherapies (i.e. paclitaxel, cyclophosphamide) in triggering T cell responses in immunogenic colon and lymphoma tumors (238). ALX148, a CD47 blocking protein, displayed high efficacy in combination with anti-PD-1, anti-human epidermal growth factor 2 (HER-2), anti-vascular endothelial growth factor receptor 2 (VEGFR-2) and anti-CD20 antibodies (known as pembrolizumab, trastuzumab, ramucirumab, rituximab respectively) and conventional chemotherapy (Paclitaxel, fluorouracil, cisplatin) in patients with malignant solid tumor and Non-Hodgkin Lymphoma (NCT03013218) (239).

Humanized IgG4 antibody (Hu5F9-G4), an anti-CD47 antibody, showed combinatorial effect with chemotherapy azacitidine in leukemia stem cells (NCT03248479) (240).

Zoledronic acid represents a double effects drug affecting both TAMs in liver cancer infiltration and decreasing tumor growth in CSCs-derived cervical cancer (241, 242). Zoledronic acid has been chosen for phase III clinical trials aiming at the prevention of bone metastasis in late-stage lung cancer patients (NCT02622607).

Of note, another innovative target is represented by myeloid-epithelial-reproductive tyrosine kinase (MERTK), a tyrosine kinase receptor discovered both in TAMs and several malignancies. MERTK, on TAMs surface, binds to the “eat-me” signal presented on apoptotic cells, activating a biological process known as “efferocytosis”. It drives the shift of macrophages to the pro-tumoral immunosuppressive M2 phenotype (243). MERTK is also overexpressed in cancer cells and is directly correlated to CSC maintenance in glioblastoma multiforme (244). The block of the MERTK signaling pathway represents a promising therapeutic strategy able to have a bidirectional effect both on TAMs and CSCs. Additionally, the administration of the agonist anti-CD40 regulates the activation of the TAM receptor CD40. Anti-CD40 mimics the homonymous ligand physiologically produced by T cells, and it leads to the reprogram of TAMs into anti-cancer macrophages with the establishment of immune surveillance (179, 245, 246). Accordingly, NG-350A, an adenoviral vector encoding for an anti-CD40 monoclonal antibody directed against tumor cells has been used to remodel the immunosuppressive TME. Interestingly, an ongoing phase I trial is investigating its systemic intravenous infusion alone or as a combinatorial treatment with pembrolizumab (NCT05165433) or chemoradiotherapy/radiotherapy (NCT06459869) in patients with advanced epithelial tumors, in particular locally advanced rectal cancer (LARC) (247). Lastly, TAMs reprogramming involves different specific biological sensors for ectopic nucleic acids such as (stimulator of interferon response cGAMP interactor (STING) and some members of toll-like receptors family (TLRs), such as TLR3, TLR7 and TLR8. The design of several synthetic compounds, which regulate these receptors on TAMs endosomal compartments, induces the activation of NF-kappa-B signaling and the consequent release of several immunostimulatory cytokines, including type I interferon (IFN-1), the master regulator of anti-cancer immunity (248–250).

As discussed above, TAMs can create an immunosuppressive TME to facilitate CSCs spreading and progression. Accordingly, the specific TAMs Inhibitor of DNA Binding 1 (ID1) + subpopulation can interact with STAT1 to localize it in the cytoplasm and inhibiting its nuclear translocation for Plasminogen activator inhibitor 2 (SERPINB2) and CCL4 transcription (251). These two factors are responsible for cancer stemness inhibition and CD8+ T cell recruitment (251). Shang et al. demonstrated that ML323 administration reduced ID1 affecting CSCs and increasing CD8+ T cells infiltration (251). In addiction ML323 treatment showed a synergistic effect with both anti-CTLA-4 antibody and 5-fluorouracil (5-FU) alone and in combination, in a colon cancer preclinical model (251). Despite the efforts made in researching therapeutic treatments to address the complex communication between TAMs and CSCs, much remains unresolved and requires further investigation and studies. The main preclinical models and clinical trials targeting CSCs-TAMs axis are summarized in **Tables 2**, **3**, respectively.

**Table 2:** Preclinical studies targeting CSCs-TAMs interactions.

Target	Target cell	Drug name or shRNA	Cancer subtype	Pre-clinical model	Reference
POSTN	CSCs	shRNA	GBM	xenograft	(189)
CCL5	TAMs	shRNA	Prostate	xenograft	(211)
SRC/CD155/MIF	TAMs	Dasanitib	NSCLC	xenograft	(232)
CD47	CSCs	B6H12	Solid tumors	xenograft	(233)
CD47	CSCs	B6H12+ paclitaxel, cyclophosphamide	Solid tumors	xenograft	(238)
MEK1-2 AKT1	CSCs	Zoledronic acid	Cervical cancer	xenograft	(241)
ID1	TAMs	ML323 + 5-FU (and/or ipilimumab)	Colorectal cancer	syngeneic	(251)

Table summarizing the most recent pre-clinical studies investigating the interaction between CSCs and TAM in different cancer subtypes.

TAMs, tumor-associated macrophages; CSCs, cancer stem cells; POSTN, Periostin; shRNA, short hairpin RNA; CCL5, Chemokine (C-C motif) ligand 5; GBM; glioblastoma multiforme; SRC, SRC Proto-Oncogene, Non-Receptor Tyrosine Kinase; CD155, cluster of differentiation 155; MIF, macrophage inhibitory factor; NSCLC, Non-Small Cell Lung Cancer; CD47, cluster of differentiation 47; MEK1-2, MAPK/ERK Kinase 1-2; AKT1, AKT serine/threonine kinase 1; 5-FU, 5-fluorouracil; ID1, Inhibitor of DNA Binding 1.

**Table 3:** Clinical trials targeting CSCs-TAMs interactions.

Target molecule	Target cell	Drug name	Combinational treatment	Cancer subtype	Clinical trial	Phase	Reference
IL-6R	CSCs	Tocilizumab	Nivolumab/Ipilimumab	Unresectable late-stage melanoma	NCT03999749	II	(229)
IL-8R (CXCR1)	CSCs	Reparixin	Paclitaxel	Breast cancer	NCT02001974	Ib	(231)
CD47	CSCs	Hu5F9-G4	Single agent	Advanced solid tumors	NCT02216409	I	(235)
CD47	CSCs	CC-90002	Rituximab	Advanced solid and Hematological cancers	NCT02367196	I	(237)
CD47	CSCs	Evorpacept (ALX148)	Pembrolizumab, Trastuzumab, Rituximab, Ramucirumab+ Paclitaxel, 5-FU+Cisplatin	Advanced solid tumors and lymphoma	NCT03013218	I	(239)
CD47	CSCs	Magrolimab (Hu5F9-G4)	Azacitidine	Hematological malignancies	NCT03248479	I	(240)
CD40	TAMs CSCs	NG-350A	Pembrolizumab	Metastatic epithelial	NCT05165433	Ia/Ib	(247)
CD40	TAMs CSCs	NG-350A	Capecitabine, radiotherapy	Locally advanced rectal cancer (LARC)	NCT06459869	Ib	(247)

Table summarizing the most recent clinical trials developed to target the CSCs-TAMs crosstalk.

TAMs, tumor-associated macrophages; CSCs, cancer stem cells; IL-6R, interleukin 6 receptor; IL-8R, interleukin 8 receptor; CD47, cluster of differentiation 47; CD40, cluster of differentiation 40; Hu5F9-G4 (5F9), humanized IgG4 antibody; 5-FU, 5-fluorouracil; LARC, Locally advanced rectal cancer.

## Advanced bioinformatics techniques based on single-cell and spatial transcriptomics focused on dissecting the role of TAMS

In the next paragraphs we summarize recent discoveries, enabled by advanced bioinformatics techniques including single-cell RNA-sequencing (scRNA-seq), spatial transcriptomics and trajectories analyses, to study TAMs in cancer progression (252). These techniques have provided crucial insights into the interactions in the TME, highlighting the pivotal role of TAMs in promoting cancer progression, influencing tumor growth, metastasis, and modulating therapeutic responses (252). TAMs exhibit functional plasticity, adopting pro- or anti-tumorigenic roles depending on environmental cues (252). The integration of scRNA-seq and spatial transcriptomics, has facilitated the dissection of TAM trajectories, signaling pathways, and their interactions with other TME components, including CSCs the dissection of TAM trajectories, signaling pathways, and their interactions with other TME components, including CSCs (252). Recent advances have revealed the dynamic interplay between TAMs and CSCs (253). The plasticity of TAMs, influenced by factors such as cytokines, chemokines, and direct cellular interactions, plays a key role in tumor dynamics (252). This review focuses on the transformative impact of bioinformatics in understanding TAM trajectories and signaling within the TME, with an emphasis on their potential for novel therapeutic interventions (252). These bioinformatics techniques are great tools for analyzing all kinds of cells, but in this review, we will focus on applications and studies for the role of TAMs. An overview regarding the bioinformatic tools to specifically study TAMs is reported in **Table 4**.

**Table 4:** Bioinformatic tools to study TAMs.

Single-cell RNA sequencing: TAM heterogeneity and functional states	
**Step**	**Best Tools**
Quality Control and Filtering	**Seurat**, Scanpy
Normalization	**Scran**, DESeq2
Clustering	Louvain, **Leiden**
Differential Expression Analysis	edgeR, **DESeq2**, MAST
Trajectory Inference	**Monocle**, Slingshot
Spatial transcriptomics: spatial organization and interactions within TME	
**Step**	**Best Tools**
Data Preprocessing	**Space Ranger**, SAW, starfish
Comprehensive Analysis	**Seurat**, Scanpy, Giotto, STUtility, Squidpy
Dimensionality Reduction and Clustering	PCA, t-SNE, **UMAP**, BayesSpace, SC-MEB, SpaGCN, STAGATE
Deconvolution and Cell Typing	**RCTD**, SPOTlight
Spatial Data Integration	Regression-based models, **deep learning approaches**
Functional Analysis and Visualization	Seurat, Scanpy, **Giotto**, Squidpy
Signaling pathway analysis: TAM signaling pathways	
**Step**	**Best Tools**
Intercellular Communication Networks	**CellChat**, CellPhoneDB

Table summarizing the most innovative bioinformatic technologies employed to study TAMs and CSCs communication in different cancer histotypes. The tools in bold are selected based on the tool performance in terms of accuracy, speed, and community adoption. However, the best choice might vary depending on specific project needs and data characteristics.

TAMs, tumor-associated macrophages; CSCs, cancer stem cells; t-SNE, t-distributed stochastic neighbor embedding; PCA, principal component analysis; UMAP, uniform manifold approximation and projection.

The tools in bold are selected based on the tool performance in terms of accuracy, speed, and community adoption.

### Single cell RNA-seq

scRNA-seq has emerged as a pivotal tool for dissecting the heterogeneity of TAMs within the TME. This technology allows the analysis of gene expression at the resolution of individual cells, providing unprecedented insights into the distinct subpopulations of TAMs and their functional states. Numerous studies have highlighted significant variations in the transcriptional profiles of TAMs across different tumor types, underscoring their role in modulating the immunosuppressive landscape of the TME (133). The application of scRNA-seq in cancer research has revealed the coexistence of TAMs with pro-tumor (M2-like) and anti-tumor (M1-like) phenotypes within tumors. This duality emphasizes the functional plasticity of TAMs in cancer progression. Recent advancements have utilized scRNA-seq to trace the developmental trajectories of TAMs, identifying key signaling pathways that regulate their polarization and function. Valdes-Mora et al. demonstrated the utility of high-throughput scRNA-seq for analyzing thousands of tumor cells, including TAMs, revealing transcriptional programs associated with different TAM states, further elucidating their roles within the TME (254).

To fully leverage scRNA-seq for TAM characterization, several bioinformatics methodologies are employed (252):

Quality Control and Filtering: Tools such as Seurat (255) and Scanpy (https://scanpy.readthedocs.io/en/stable/) are commonly used to filter low-quality cells based on the number of detected genes and mitochondrial content.

Normalization: Normalization of scRNA-seq data is essential for accurate downstream analysis. Techniques like Scran or DESeq2 (256) provide effective approaches for normalization.

Clustering: To identify distinct cell populations, clustering algorithms like Louvain or Leiden are employed, allowing for robust community detection in high-dimensional datasets (257).

Differential Expression Analysis: To uncover differences in gene expression across TAM subpopulations, tools such as edgeR (258), DESeq2 (256), or MAST (259) are frequently used, depending on the analysis framework.

scRNA-seq has provided groundbreaking insights into the transcriptional diversity and functional heterogeneity of TAMs across various cancer types (260). This approach has enabled the identification of distinct TAM subtypes, each contributing differently to tumor immunity and progression. Specifically, studies in breast cancer have delineated M1-like and M2-like TAM populations, revealing their unique roles in promoting or inhibiting tumor growth (261, 262). This emerging knowledge is crucial for the development of targeted therapies aimed at reprogramming TAMs to a more anti-tumor state, offering new avenues for therapeutic intervention in cancer. scRNA-seq represents a transformative approach in TAM research, providing high-resolution profiling of individual TAMs and enabling the identification of diverse subpopulations based on their gene expression profiles. By combining scRNA-seq with advanced bioinformatics tools, researchers can uncover the full spectrum of TAM heterogeneity and its implications for cancer progression and therapy. This method excels at revealing transcriptional heterogeneity and elucidating the cellular and molecular mechanisms underlying TAM function within the TME. However, it requires tissue dissociation, which disrupts the spatial organization of the tumor microenvironment and leads to a loss of spatial information. This limitation prevents a direct understanding of TAM interactions within their native tissue context, which is crucial for fully characterizing TAM functionality in relation to the TME.

### Spatial transcriptomics for TAM trajectories

While scRNA-seq has provided significant insights into the heterogeneity of TAMs, it lacks spatial resolution, which is crucial for understanding their interactions within the TME. Spatial transcriptomics bridges this gap by integrating gene expression data with spatial information, allowing for the precise mapping of TAM distribution and organization within tumor tissues. This spatial context is essential for capturing the complexity of TAM interactions with other cell types and their influence on tumor progression. Spatial transcriptomics has been extensively applied to study the spatial dynamics of TAMs across various cancer types. It has been demonstrated that TAMs located within the tumor stroma and at invasive tumor margins exhibit distinct gene expression profiles and functional states, which play a pivotal role in driving tumor progression and metastasis (263). By integrating spatial transcriptomics with scRNA-seq data, researchers can gain a more comprehensive understanding of TAM trajectories and their interactions with other cells in the TME. Recent developments in bioinformatics have facilitated the analysis of spatial transcriptomics data, from preprocessing to functional interpretation.

These advancements include:

Data Preprocessing: The initial steps of spatial transcriptomics analysis involve generating a gene expression matrix along with spatial coordinates. Tools such as Space Ranger (10X Genomics), SAW (Stereo-seq), and starfish (ISS/ISH) are widely used for data preprocessing, depending on the platform and methodology employed (264).

Comprehensive Analysis Tools: Seurat (255) and Scanpy (https://scanpy.readthedocs.io/en/stable/) are versatile tools frequently used for both scRNA-seq and spatial transcriptomics analysis. These platforms offer functionalities for filtering, normalization, and various downstream analyses. For more specialized spatial transcriptomics tasks, Giotto (265), STUtility (266), and Squidpy (267) provide extended capabilities, including advanced spatial analyses (264).

Dimensionality Reduction and Clustering: Techniques such as principal component analysis (PCA), t-distributed stochastic neighbor embedding (t-SNE), and uniform manifold approximation and projection (UMAP) are widely employed for dimensionality reduction (268). In addition, spatial-specific algorithms like BayesSpace, SC-MEB, SpaGCN, and STAGATE leverage spatial information to enhance clustering accuracy and identify spatial features (264).

Deconvolution and Cell Typing: Since sequencing data often represents aggregate signals from multiple cell types, deconvolution techniques are required to resolve individual cell-type contributions. Tools such as RCTD and SPOTlight facilitate accurate cell type identification in spatial datasets by leveraging scRNA-seq data for reference (269).

-Spatial Data Integration: Integrating scRNA-seq data with spatial transcriptomics enables comprehensive spatial characterization of gene expression patterns. Regression-based models and deep learning approaches are commonly used to reconstruct missing spatial features and enhance gene expression data (263).

Functional Analysis and Visualization: Tools like Seurat and Scanpy provide robust visualization capabilities for spatial transcriptomics data, while specialized platforms like Giotto and Squidpy allow for more detailed analyses of cellular interactions, spatial neighborhood graphs, and trajectory inference (264).

The integration of spatial transcriptomics with advanced bioinformatics tools has significantly enhanced our understanding of the spatial organization of TAMs within the TME. These methods enable the comprehensive analysis of spatial gene expression patterns, providing valuable insights into the architecture of tumor tissues and the interactions between TAMs and other cell types. These advancements are critical for identifying novel diagnostic markers and therapeutic targets, furthering our ability to design effective cancer therapies (264).

Spatial transcriptomics complements scRNA-seq by offering spatial context to gene expression data, allowing researchers to visualize TAM localization and their interactions with other cell types *in situ*. This approach has been utilized to map TAM heterogeneity across lung cancer subtypes, revealing distinct macrophage compositions that correlate with specific tumor characteristics (270). By preserving tissue architecture, spatial transcriptomics facilitates a comprehensive analysis of cellular communication and the organization of the TME, which is essential for elucidating the functional roles of TAMs in cancer progression. However, compared to scRNA-seq, spatial transcriptomics typically offers reduced sensitivity and lower coverage, particularly for detecting genes expressed at low levels, which can limit the depth of transcriptomic insights.

### Trajectory analysis

scRNA-seq has become an essential tool for studying cellular heterogeneity within tumors, enabling the characterization of distinct cell populations, including TAMs. By applying trajectory inference methods to scRNA-seq data, researchers can reconstruct the developmental pathways of individual cells based on their gene expression profiles, providing critical insights into cellular differentiation and function within the TME.

Pseudotime analysis is a widely used approach to order cells along a developmental trajectory, providing insights into their differentiation states. In the context of TAMs, pseudotime analysis has been employed to reveal the dynamic transitions of these cells as they interact with tumor cells and other components of the TME. Wang et al. elucidated the TAMs transition from pro-inflammatory to immunosuppressive phenotypes during breast cancer progression, demonstrating the utility of pseudotime analysis in understanding TAM functional changes over time (271).

Trajectory inference tools such as Monocle (272) and Slingshot (273) are commonly used to identify genes that are differentially expressed along inferred cellular trajectories. These techniques have proven valuable in uncovering key molecular pathways involved in TAM function and tumor progression. Yang et al. discovered that TAMs regulate BCSCs through a paracrine signaling pathway involving epidermal growth factor receptor (EGFR), STAT3, and SOX2, highlighting the relevance of trajectory analysis in elucidating cell-cell interactions within the TME (274).

Trajectory analysis aims to reconstruct the differentiation pathways and developmental trajectories of cells over time, offering a temporal perspective on how TAMs transition between distinct functional states. Applying trajectory inference to TAM scRNA-seq data has enabled researchers to discern how these macrophages evolve in response to tumor signals and alterations in the TME. Saelens et al. utilized trajectory analysis to gain insights into the temporal dynamics of TAM polarization during cancer progression, identifying key transitions from pro-inflammatory to immunosuppressive states (275).

The interactions between TAMs and CSCs are critical for driving tumor progression and metastasis. TAMs secrete various factors that enhance CSC properties, promoting tumor growth and resistance to therapy. Valdes-Mora et al. showed that TAM-derived cytokines, such as IL-6 and IL-10, help maintain the stemness of CSCs in breast cancer, underscoring the importance of TAM-CSC crosstalk in the TME (254).

In a glioblastoma study the authors used Monocle to trace the differentiation trajectories of TAMs, revealing changes in their functional states in response to tumor-derived signals (276). Furthermore, the integration of spatial transcriptomics with scRNA-seq allows for a more nuanced understanding of TAM interactions with other immune and tumor cells, providing a spatial and temporal view of TAM dynamics within the TME (277).

### Signaling pathways in TAMs

Advanced bioinformatics techniques have significantly enhanced the ability to identify critical signaling pathways that regulate the functions of TAMs and their impact on cancer progression. Jin et al. utilized spatial transcriptomics to uncover spatially regulated biomarkers and signaling pathways within TAM populations, providing valuable insights into their roles and functional states within the TME (264). These approaches have also elucidated specific signaling pathways that govern TAM polarization and their pro- or anti-tumoral activities. The CCL2-CCR2 signaling axis has been shown to play a pivotal role in recruiting and polarizing TAMs toward a pro-tumorigenic phenotype (278). Importantly, the inhibition of this pathway holds therapeutic potential by reprogramming TAMs toward an anti-tumor phenotype, thereby enhancing the effectiveness of cancer treatments (278). This underscores the significance of targeting TAM-specific pathways in therapeutic strategies aimed at modulating the TME.

TAMs influence a wide array of signaling pathways within the TME, directly interacting with tumor cells and other TME components to drive cancer progression. Pathways such as WNT, NOTCH, and TGF-beta, which are crucial for maintaining CSC properties and promoting tumor aggressiveness, are modulated by TAM activity (279). These pathways are critical for the survival and function of CSCs, further supporting the tumor’s growth and metastasis.

Recent studies have leveraged computational models to simulate the effects of TAMs on tumor growth, shedding light on the importance of cell-cell communication in the TME. Zhao et al. demonstrated that TAMs secrete cytokines and chemokines that enhance CSC survival and drive tumor progression (138). These computational insights highlight the complex interactions within the TME that are essential for tumor evolution. Emerging bioinformatics tools such as CellChat (https://github.com/sqjin/CellChat) or CellPhoneDB (280) have proven effective in analyzing intercellular communication networks, providing a deeper understanding of ligand-receptor interactions that regulate TAM and CSC dynamics. Through the application of such tools, researchers have been able to map intricate communication networks between TAMs and other cells in the TME. These findings offer opportunities to identify novel therapeutic targets aimed at reprogramming TAMs toward an anti-tumor phenotype, potentially improving cancer treatment outcomes (281). Overall, integrating bioinformatics approaches with experimental data has been pivotal in uncovering the signaling pathways that govern TAM activity. These insights offer potential strategies for therapeutic interventions aimed at altering TAM function and modulating the TME to halt tumor progression. An overview of the most important discoveries made about the CSCs-TAMs axis by advanced bioinformatic technologies in different cancer histotypes is summarized in **Table 5**.

**Table 5:** Discoveries in TAMs-CSCs axis research by bioinformatic approaches.

Technique	Main Discovery	Cancer Type	Focus	Reference
single-cell RNA sequencing, spatial transcriptomics	The co-location of CSCs and SPP1+ macrophages in a hypoxic region correlates with poor prognosis in HCC.	hepatocellular carcinoma	CSC	(283)
single-cell RNA sequencing, spatial transcriptomics	A distinct glioma stem cell population was identified, characterized by high proliferative potential and an enrichment of E2F1, E2F2, E2F7, and BRCA1 regulons, with implications for tumor growth and patient outcomes.	glioma	TAMs	(284)
single-cell RNA sequencing	scRNAseq allows for a detailed characterization of the TME in HNSCC, enhancing understanding of cancer biology and treatment responses.	head and neck squamous cell carcinoma	CSC	(285)
single-cell RNA sequencing	scRNA-seq provides insights into the tumor microenvironment and intratumor heterogeneity in gastric cancer, revealing the roles of various immune cells and their interactions.	gastric cancer	TAMs	(286)
single-cell RNA sequencing, spatial transcriptomics	POSTN + cancer-associated fibroblasts are associated with immune suppression and poor prognosis in non-small cell lung cancer.	non-small cell lung cancer	CSC	(287)
single-cell RNA sequencing, spatial transcriptomics	GSDensity allows pathway-centric interpretation and dissection of single-cell and spatial transcriptomics data, revealing novel cell-pathway associations and creating a pan-cancer ST map.	various tumor types	CSC	(288)
single-cell RNA sequencing	Macrophage-naive CD4 + T cell interaction significantly affects the cancerous state in liver carcinoma.	liver carcinoma	TAMs	(289)
single-cell RNA sequencing, spatial transcriptomics	Defines the cellular composition and architecture of cutaneous squamous cell carcinoma (cSCC), identifying tumor subpopulations and their spatial interactions.	cutaneous squamous cell carcinoma	TAMs	(290)

Table summarizing the most important discoveries made about the CSCs-TAMs axis by advanced bioinformatic technologies in different cancer histotypes.

TAMs, tumor-associated macrophages; CSCs, cancer stem cells; SPP1, secreted phosphoprotein 1; POSTN, periostin; HNSCC, head and neck squamous cell carcinoma; BRCA1, BRCA1 DNA repair associated; HCC, hepatocellular carcinoma; TME, tumor microenvironment; E2F1, E2F transcription factor 1; E2F2, E2F transcription factor 2; E2F7, E2F transcription factor 7.

## Concluding remarks

The direct and indirect mechanisms of interaction between TAMs and CSCs are crucial for cancer development, for the regulation of the metastatic niche, and ultimately for the formation of metastatic lesions. TAMs can establish with CSCs an intricate complex communication in fueling different aspects of cancer progression: i) direct ligand-receptor interaction; ii) indirect: TAMs-secreted chemokines/cytokines/exosomes foster CSC stemness, metastatization and chemoresistance respectively; CSC-derived exosomes reprogram TAM toward M2 immunosuppressive phenotype.

Both scRNA-seq and spatial transcriptomics offer unique advantages and limitations in the study of TAMs, with the choice between these techniques largely dependent on the specific research question. In summary, scRNA-seq is advantageous for detailed molecular profiling and understanding TAM heterogeneity, while spatial transcriptomics is better suited for exploring TAM spatial distribution and interactions within the TME. An integrated approach combining both methods would provide a more comprehensive understanding of TAM biology by capturing both transcriptional diversity and spatial dynamics.

The cutting-edge single cell-based and spatial transcriptomics technologies may shed new lights on the specific role of TAMs in promoting CSCs and cancer development and may help to design innovative therapeutic approaches aimed at disrupting this cross talk.

## Glossary

5-FU: 5-fluorouracil
ABC: ATP-binding cassette
ADAMTS6: ADAM metallopeptidase with thrombospondin type 1 motif 6
ANXA3: annexin A3
AKT1: AKT serine/threonine kinase 1
ATF2: activating transcription factor 2
BCSCs: breast cancer stem cells
BRCA1: BRCA1 DNA repair associated
BTN3A3: butyrophilin subfamily member A3
CAFs: cancer-associated fibroblasts
CAR-T: chimeric antigen receptor T cell
CCL1: chemokine (C-C motif) ligand 1
CCL2: chemokine (C-C motif) ligand 2
CCL5: chemokine (C-C motif) ligand 5
CCL18: chemokine (C-C motif) ligand 18
CCL20: chemokine (C-C motif) ligand 20
CCR2: C-C chemokine receptor type 2
CD11b: cluster of differentiation 11b
CD44: cluster of differentiation 44
CD47: cluster of differentiation 47
CD80: cluster of differentiation 80
CD90: cluster of differentiation 90
CD155: cluster of differentiation 155
CHAC1: ChaC Glutathione Specific Gamma-Glutamylcyclotransferase 1
CRC: colorectal cancer
CRLM: colorectal liver metastasis
CSCs: cancer stem cells
CSF1: colony stimulating factor 1
CTL: cytotoxic T lymphocytes
CTLA-4: cytotoxic T lymphocyte associated protein 4
CXCL5: C-X-C motif chemokine ligand 5
CXCL12: C-X-C motif chemokine ligand 12
CXCL13: C-X-C motif chemokine ligand 13
DC: dendritic cell
DDR: DNA damage response
DTCs: Disseminated tumor cells
E2F1: E2F transcription factor 1
E2F2: E2F transcription factor 2
E2F7: E2F transcription factor 7
ECM: extracellular matrix
EIF4EBP1: Eukaryotic translation initiation factor 4E-binding protein 1
EGFR: epidermal growth factor receptor
EMT: epithelial-mesenchymal transition
EOCCs: epithelial ovarian cancer cells
EPHA4: ephrin type-a receptor 4
ERK1/2: extracellular regulated kinase 1/2
ET-1: endothelin 1
FASL: FAS ligand
FGF: fibroblast growth factor
FGF7: fibroblast growth factor 7
FGF9: fibroblast growth factor 9
FPR2: formyl peptide receptor 2
FYN: FYN proto-oncogene
GBM: glioblastoma multiforme
GZMB: granzyme B
GSCs: glioblastoma stem cells
HAS2: enzyme hyaluronan synthase 2
hCAP-18/LL-37: immunomodulatory cationic antimicrobial peptide 18
HCC: hepatocellular carcinoma
HER-2: human epidermal growth factor 2
HHCSCs: human hepatocellular carcinoma stem cells
HIFs: inducing factors of hypoxia
HIF1A: hypoxia inducible factor 1 subunit alpha
HIF1B: hypoxia inducible factor 1 subunit beta
HIF2A: hypoxia inducible factor 2 subunit alpha
HIF3A: hypoxia inducible factor 3 subunit alpha
HLA: human leukocyte antigen
HLA-G: human leukocyte antigen G
NANOG: homeobox protein NANOG
HNSCC: head and neck squamous cell carcinoma
HSCs: hematopoietic stem cells
Hu5F9-G4 (5F9): humanized IgG4 antibody
ID1: Inhibitor of DNA Binding 1
IFN-1: type I interferon
IFNG: interferon-gamma
IL-2: interleukin-2
IL-6: interleukin-6
IL-8: interleukin-8
IL-10: interleukin-10
IL-12: interleukin-12
IL-17: interleukin-17
IL-6R: interleukin-6 receptor
IL-8R: interleukin-8 receptor
ISG15: Interferon-stimulated gene 15
JAK-STAT: Janus kinase/signal transducers and activators of transcription
KIR2DL4: killer cell immunoglobulin like receptor, two Ig domains and long cytoplasmic tail 4
KLF4: KLF transcription factor 4
LARC: Locally advanced rectal cancer
LOX: Lysyl oxidase
LSCC: lung squamos cell carcinoma
LSECtin: liver and lymph node sinusoidal endothelial cell C-type lectin
MAMs: metastasis-associated macrophages
MAPK: mitogen-activated protein kinase
MDR: multidrug resistance
MDSCs: myeloid-derived suppressor cells
MEK1-2: MAPK/ERK kinase 1-2
MERTK: myeloid-epithelial-reproductive tyrosine kinase
MetCSCs: metastatic cancer stem cells
MFGE8: milk fat globule-EGF factor 8
MHC-I: major Histocompatibility Complex Class I
MICA: major Histocompatibility Complex Class I chain-related protein A
MICB: major Histocompatibility Complex Class I chain-related protein B
MIF: macrophage inhibitory factor
miR-221-3p: microRNA-221-3p
miR-934: microRNA-934
MMIC: malignant melanoma initiating cells
MMP-2: matrix metalloproteinases 2
MMP-9: matrix metalloproteinases 9
MTOR: mammalian target of rapamycin
MSCs: mesenchymal stromal cells
MYC: MYC proto-oncogene protein
NF-kappa-B: nuclear factor-kappa B
NK: natural killer cells
KLRC1: killer cell lectin like receptor C1
KLRK1: killer cell lectin like receptor K1
NSCLC: non small cell lung cancer
NSCLCCSCs: non-small cell lung cancer stem cells
OCT4: octamer-binding transcription factor 4
OSCC: oral squamous cell carcinoma
P2X7R: P2X purinoceptor 7 receptor
PCA: principal component analysis
PD-1: programmed death protein 1
PD-L1: programmed death-ligand 1
PDAC: pancreatic ductal adenocarcinoma
PDGF: platelet-derived growth factor
PDGFB: platelet-derived growth factor B subunits
PGE2: prostaglandin E2
PI3K: phosphatidylinositol 3-kinase
POLN: DNA polymerase nu
POSTN: periostin
PPAR: peroxisome proliferator-activated receptor
PRF1: perforin 1
PTEN: phosphatase and tensin homolog
PTN: pleiotrophin
PTPRZ1: tyrosine phosphatase receptor type Z1
ROS: reactive oxygen species
SCCHN: squamous cell carcinoma of head and neck
scRNA-seq: single-cell RNA-sequencing
SERPINB2: Plasminogen activator inhibitor 2
SHH: Sonic hedgehog
SIRPA: signal-regulatory protein alpha
SOX2: SRY-Box transcription factor 2
SPP1: secreted phosphoprotein 1
SRC: SRC proto-oncogene, non-receptor tyrosine kinase
STAT3: signal transducer and activator of transcription 3
STING: stimulator of interferon response cGAMP interactor 1
t-SNE: t-distributed stochastic neighbor embedding
TAMs: tumor-associated macrophages
TGFB1: transforming growth factor-beta-1
THBS1: thrombospondin 1
TLRs: toll-like receptors
TME: tumor microenvironment
TNBC: triple negative breast cancer
TNF-alpha: tumor necrosis factor-alpha
TRAIL: TNF-related apoptosis-inducing ligand
Tregs: regulatory T cells
UMAP: uniform manifold approximation and projection
VCAM: vascular endothelial cell adhesion molecule
VEGF: vascular endothelial growth factor
VEGFR-2: vascular endothelial growth factor receptor 2
VM: vasculogenic mimicry
WNT: wingless-related integration site
ZEB1: Zinc finger E-box binding homeobox 1
